# Structural considerations of vitamin D signaling

**DOI:** 10.3389/fphys.2014.00191

**Published:** 2014-06-06

**Authors:** Ferdinand Molnár

**Affiliations:** Faculty of Health Sciences, School of Pharmacy, Institute of Biopharmacy, University of Eastern FinlandKuopio, Finland

**Keywords:** VDR, crystal structure, molecular dynamics, molecular mechanism, cofactors, response elements, ligand-binding

## Abstract

Crystal structures represent the static picture in the life of a molecule giving a sneak preview what it might be in reality. Hence, it is very hard to extrapolate from these photos toward dynamic processes such as transcriptional regulation. Mechanistically VDR may be considered as molecular machine able to perform ligand-, DNA- and protein recognition, and interaction in a multi-task manner. Taking this into account the functional net effect will be the combination of all these processes. The long awaited answer to explain the differences in physiological effects for various ligands was one of the biggest disappointment that crystal structures provided since no substantial distinction could be made for the conformation of the active VDR-ligand complexes. This may have come from the limitation on the complexity of the available ligand-VDR structures. The recent studies with full length VDR-RXRα showed somewhat more comprehensive perspective for the 3D organization and possible function of the VDR-RXRα-cofactor complex. In addition to *in vitro* approaches, also computational tools had been introduced with the aim to get understanding on the mechanic and dynamic properties of the VDR complexes with some success. Using these methods and based on measurable descriptors such as pocket size and positions of side chains it is possible to note subtle differences between the structures. The meaning of these differences has not been fully understood yet but the possibility of a “butterfly effect” may have more extreme consequences in terms of VDR signaling. In this review, the three functional aspects (ligand-, DNA- and protein recognition, and binding) will be discussed with respect to available data as well as possible implication and questions that may be important to address in the future.

## Introduction

One way of understanding life at molecular level is to obtain the three-dimensional (3D) structures of the molecules. Such structural views represent a static picture in the life of a molecule giving a sneak preview what it might be in reality. For the understanding of the functional implication of vitamin D (VD) signaling it is also important to look at various structural complexes of the vitamin D receptor (VDR), which may outline its possible dynamics and mechanics. VDR is able to perform the ligand-, DNA- and protein recognition, and interaction in a multi-task manner thus can be viewed as molecular machine which will regulate gene expression with the combination/sum of all these particular functions.

Before the year 2000 people in the VD field were only guessing how may the 3D structure of VDR look like. Some implications were coming from already known crystal structures e.g., receptors for retinoids such as retinoid X receptor (RXR) (Bourguet et al., 1995) and retinoic acid receptor (RAR) (Renaud et al., 1995). Not until exactly 14 years ago the structure of VDR-1α,25-dihydroxyvitamin D_3_ (1,25D_3_) complex has been solved (Rochel et al., 2000) and a long journey started in understanding the binding of various VDR analogs and the structure-based analog design. Within the first 5 years more structures have been solved (Tocchini-Valentini et al., 2001, 2004; Eelen et al., 2005) but the long awaited answer to explain the differences in physiological effects for various ligands was one of the biggest disappointment that crystal structures provided since no substantial distinction could be made for the conformation of the active VDR-ligand complexes.

Compared to the beginning of the last decade a huge number of X-ray crystal structures are available for VDR. In detail, there are VDR LBDs from three different species *H. sapiens* (34), *R. norvegicus* (40) and *D. rerio* (13) and four DBD-DNA complexes from *H. sapiens*. The basic information about these complexes is summarized in Table 1. This data makes also possible to analyze orthologous molecules with reflection to functional and structural differences. However, to understand this aspect well it would be beneficial to have more data from numerous organisms. In this review evolutionary aspects and species-specific difference will be not discussed in depth. More space will be given to general domain organization, binding mode of natural ligands and recognition of DNA by VDR. Some data coming from molecular dynamics (MD) simulations will be also discussed since this approach represents a compromise in obtaining 3D structural models and have been proven to be well aligned with the wet lab data. At last, recent data from small-angle X-ray scattering (SAXS), cryo-electron microscopy (cryo-EM) and H/D exchange (HDX) experiments will be discussed with some perspectives highlighted.

**Table 1:**
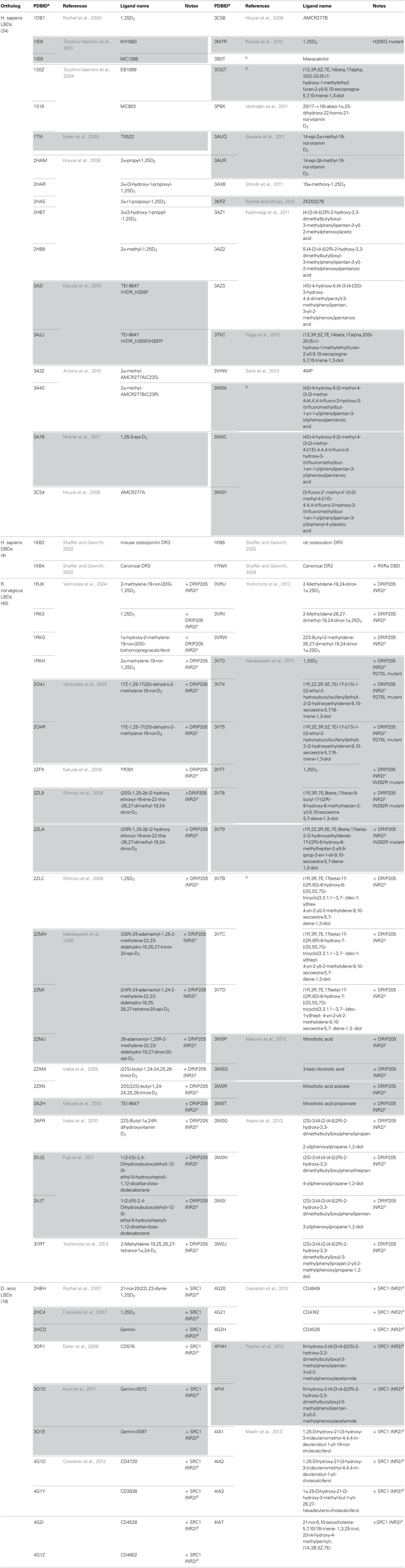
**List of crystal structure data available for LBD and DBD of VDR (source: www.pdb.org)**.

^a^Protein Data Bank identifier.

^b^Unpublished structure.

^c^Synthetic peptide corresponding to NR2 Box of DRIP205/TRAP220/MED1.

^d^Synthetic peptide corresponding to NR2 Box of SRC1.

## What do VDR structures tell us?

**Figure 1:**
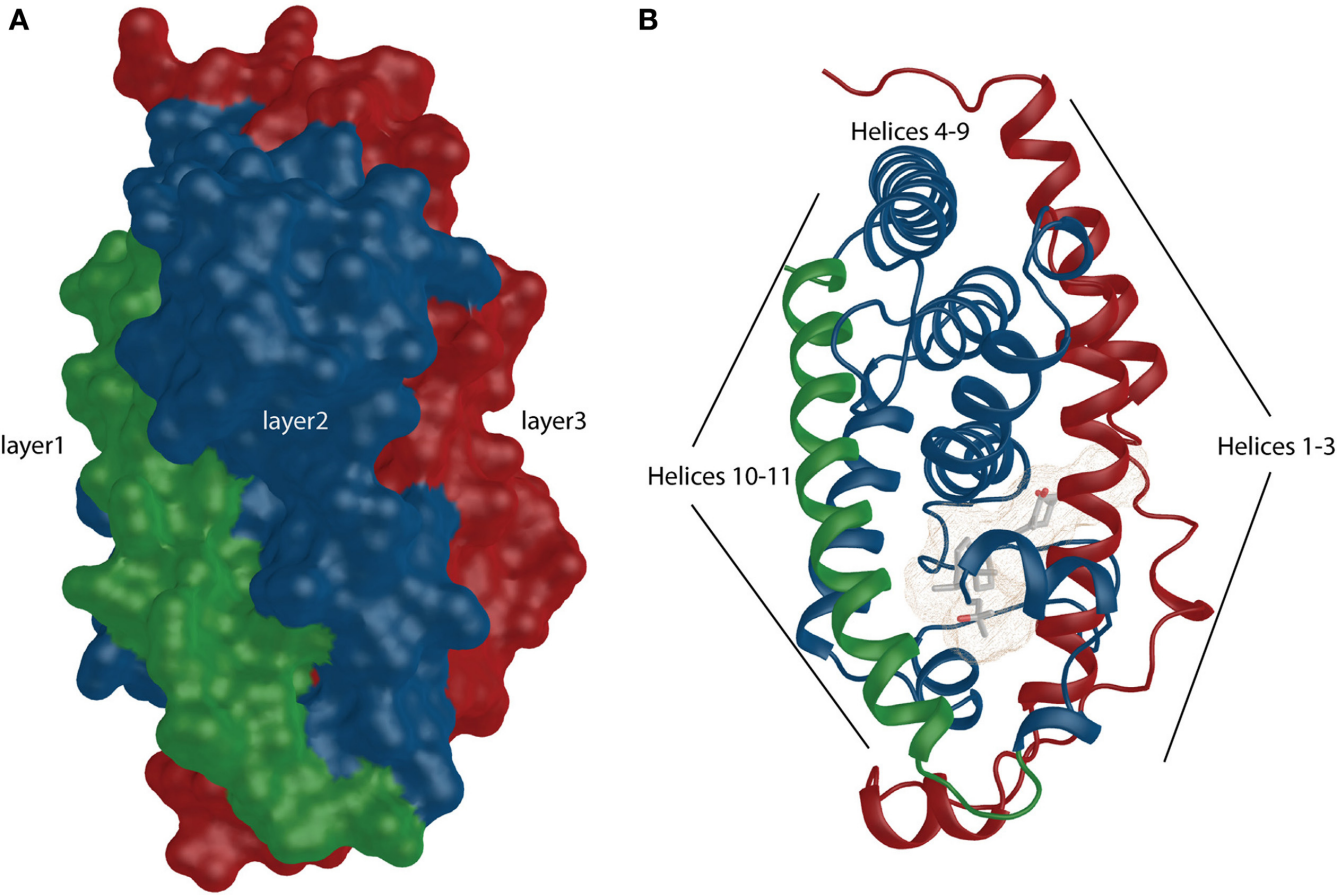
**VDR shows similarity to canonical NR structural organization. (A)** The overall surface depiction of the VDR showing the three layers sandwich-like molecule where the layers are highlighted in green, blue and red. **(B)** Numbered helices belonging to different layers are shown and they are highlighted similarly as in surface representation.

**Figure 2:**
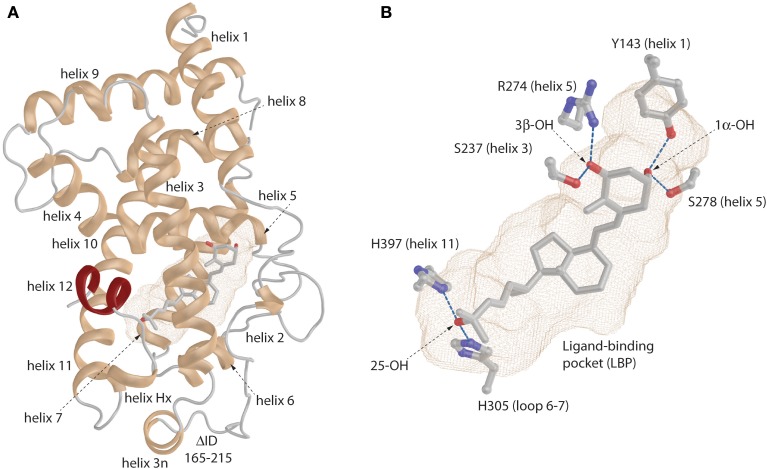
**General view of the VDR. (A)** Ribbon representation of the VDR with annotated helices. The very last helix (helix 12) is highlighted in red color. **(B)** The binding mode of the 1,25D in the ligand-binding pocket (LBP). The important anchoring residues and their location in the VDR structure is depicted. Hydrogen bonds are shown in blue (for details see the text).

From functional and structural organization point of view VDR is formed by DNA-binding domain (DBD:24–89 aa; domain C), ligand-binding domain (LBD:126–427 aa; domain E), a connective hinge (domain D) between them and a short A/B domain located at the N-terminus. Compare to RXR it completely lacks the F domain, which is the very last part of the LBD after helix 12. The LBD is formed by a three layer anti-parallel α-helical sandwich (Figures 1A,B highlighted in green, blue and red). Based of the particular structure it contains all together 11–13 α-helices (Li et al., 2003) (Figure 2A). The internal structure of the LBDs of the respective nuclear receptors (NRs) shows a high similarity with specialized diversity based on functional properties of the particular receptor. In VDR, the LBD is responsible for active ligand recognition and interaction with partnering proteins such as coregulators, and RXR to form the functionally active RXR-VDR heterodimer. In particular, helices 3, 4, and 10–12 are involve in the interaction with protein partners. Interestingly, to date all solved VDR crystal structures show very ubiquitous and conserved organization of the of overall structural fold not reflecting the divergent nature of the bound natural or synthetic ligands. What are in fact the differences in structures that reflect various physiological effects of the particular ligands? Allegedly there will not be a simple answer to this question since we may face the limitation on the complexity of the available ligand-VDR structures or have to allow the possibility that the subtle differences between the structures may cause a “butterfly effect” that have more extreme consequences in terms of VDR signaling than initially thought. By all means there are important differences in the metabolism of various synthetic ligands and a possible unique coactivator recruitment may also play its role. However, none of these possibilities can be fully explored using the available VDR crystal structures. Nevertheless, what we may agree on is that all VDR crystal structures show agonistic conformation, surprisingly even in case of antagonists, that is canonically represented by a closed conformation of the helix 12 providing a docking platform for the recruitment of coactivators. This may be due to the shifted equilibrium that drives VDR for closed helix 12 with minimal energy conformation. In addition, the VDR structures do tell us the binding mode, anchoring points and subtle changes in the position of residues that may be effectively used for *de novo* design of superagonist such as AMCR277A (PDBID:3CS4) (Hourai et al., 2008). The frequently mentioned subtle changes that are characteristic for the ligand-binding pocket (LBP) may be further analyzed and can explain some of the binding differences between various ligands in correlation to their functional and biological properties.

## The inner circle: looking into the VDR pockets

The “lower part” of the LBDs of all ligand-activated NRs contains a LBP which volume size range between 400 and 1400 Å^3^ (Figures 2A,B light-brown mesh) (Nagy and Schwabe, 2004). It is not quite different in case of VDR where LBP primarily serves for effective recognition of various natural ligands such as 1,25D_3_ and its metabolites or bile acids. In addition, this is one of the most important parts to modulate VDR's activity via various synthetic compounds. The VDR pocket can be placed in the middle range of the volume scale showing rather high dynamic plasticity toward various ligands.

**Figure 3:**
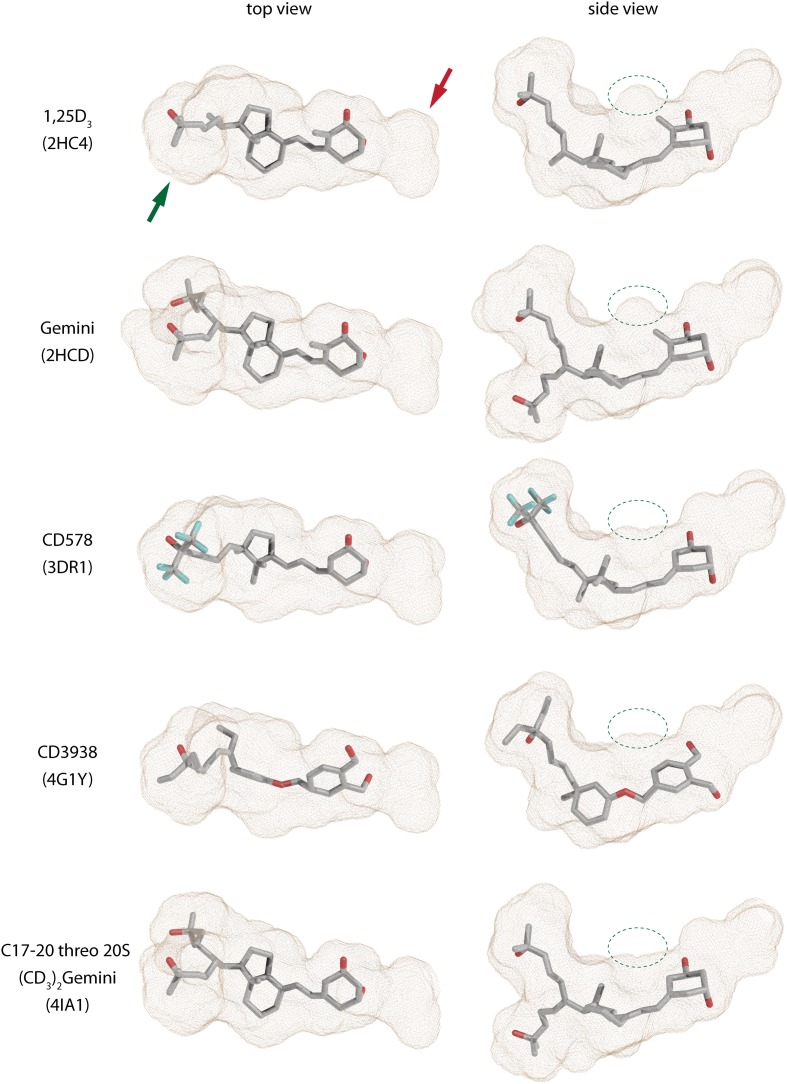
**Shapes of the zebrafish VDR ligand-binding pockets (LBPs) with various compounds (source PDBID: 2HC4, 2HCD, 3DR1, 4G1Y, and 4IA1)**.

The first VDR crystal structure confirmed the conserved contact or anchoring points for the interaction of VDR with 1,25D_3_ (Rochel et al., 2000) (Figure 2B). The residues involved in the positioning of the 1,25D_3_ in the LBP are Y143 (helix 1) and S278 (helix 5) that contact the 1,25D_3_1α-hydroxyl (OH) group, S237 (helix 3) and R274 (helix 5) contact 3β-OH, and H305 (loop 6–7) and H397 (helix 11) interact with 25-OH. In most of the cases if these anchoring point are disrupted a decrease in the activation potential of the ligand can be observed. Besides these residues the interior surface of the VDR LBP is formed of about 40 mostly non-polar amino acids. An interesting approach is to look how the LBP is changed upon ligand-binding especially its volume or how much volume (%) is occupied by the ligand. A rather straightforward example is the comparison of the 1,25D_3_ and MC1288 LBPs. Although the volumes of these ligands are highly comparable 434 Å^3^ and 427 Å^3^ (probe radius 1.9 Å), respectively, the volumes of the LBP show slight differences 776 Å^3^ and 643 Å^3^ (same probe radius) leading to ratio of 56 and 66% of ligand occupancy of the LBP volume (Molnár et al., 2006). When a ligand occupancy is higher compared to 1,25D_3_ then it increases the stability of VDR. This factor holds true for ligands that show high structural similarity and binding mode to 1,25D_3_. It seems to some extend that the ratio of the ligand to LBP volume can be a good descriptor of the ligands' activation potential. In addition, the actual shape of the cavity also reflects differences in the binding of various ligands which is illustrated in Figure 3. There are slight differences in the shapes of the LBP depending on the bound ligand. The red arrow shows the part where the shape is conserved well. Topologically this is the place where the 1,25D_3_ A-ring is located e.g., helices 1, 3, and 5. The more plastic part is the one where the 1,25D_3_ side chain is found with helices 6, 7, and 11. This region shows the highest variation between the ligand-bound structures and is indicated with green arrow Figure 3. In addition, small changes can be also detected for instance the part highlighted with green circle. Very interesting in the difference between the two Gemini structures 2HCD and 4IA1, where the largest difference for the two side chains is that “C17–20 threo 20S—Gemini” has the hydrogens in its methyl groups substituted with deuterium. Interestingly, in this case the pocket shows more compact shape in the region with the double side chains and the volume of the location highlighted with green circle is decreased, which may be due to modification of the A-ring. Another interesting question is the maximum volume to which the VDR LBP can be stretched. MD simulations showed that by docking a Gemini with fluorinated methyls groups, (CF3)_2_-Gemini, the LBP could be expanded by 1/3 of the 1,25D_3_ LBP volume. In proportion to this, the compound's volume in the pocket is also increased about 30%.

An appealing view opens up when a comparison of the VDR structure with one of its closest relative pregnane X receptor (PXR) is made (Watkins et al., 2001). As for VDR there are plenty of PXR crystal structures available and it can extend its LBP to very large volume (~1400 Å^3^) to accommodate various compounds. The published VDR structures lack the insertion domain (Δ166–216 aa), but even without it the LBP as discussed earlier is as large as 700–800 Å^3^ with MD simulations showing that it can expand beyond 1000Å^3^, which is already comparable to PXR's LBP. This may suggest that VDR is able to accommodate variety of other compounds in addition to 1,25D_3_ such as LCA/3-keto-LCA (Makishima et al., 2002). The later have been crystallized with rVDR and will be discussed in this review. The indications for binding additional compounds besides 1,25D_3_ are coming also from *P. marinus* (sea lamprey) where despite of lack of the calcified skeleton and teeth it may serve as a xenobiotic activator for detoxification by regulating P450 enzymes (Whitfield et al., 2003; Krasowski et al., 2005). However, it is yet to be determined whether there are existing other ligands that bind to VDR. One part of the LBD, which may allow the binding of these compounds is the insertion domain. Although its clear functional role has not been identified and it seems that it is not directly required for the binding of the 1,25D_3_ (Rochel et al., 2001), it may play some other roles. A mutation C190W was reported in patients that results in loss of 1,25D_3_ binding (Malloy et al., 1999), though this may be due to the disruption of the VDR structure by introducing a large bulky tryptophan residue. Secondly, the homologous part of PXR (142–431 aa) especially the occurrence of the two β-strands and the associated coiled regions are responsible for the expandability of the PXR's LBP thus an analogous role cannot be out ruled in case of VDR as well. There has been also proposed that an alternative pocket, which can be considered as an enlargement of the original pocket, is formed in the VDR that extends toward the helix 2/β-sheet region of the LBD (Mizwicki et al., 2004). Especially this can be observed with covalently locked 1,25D_3_-derived compounds such as 1,25(OH)_2_-lumisterol, which has been showed by *in silico* docking studies.

**Figure 4:**
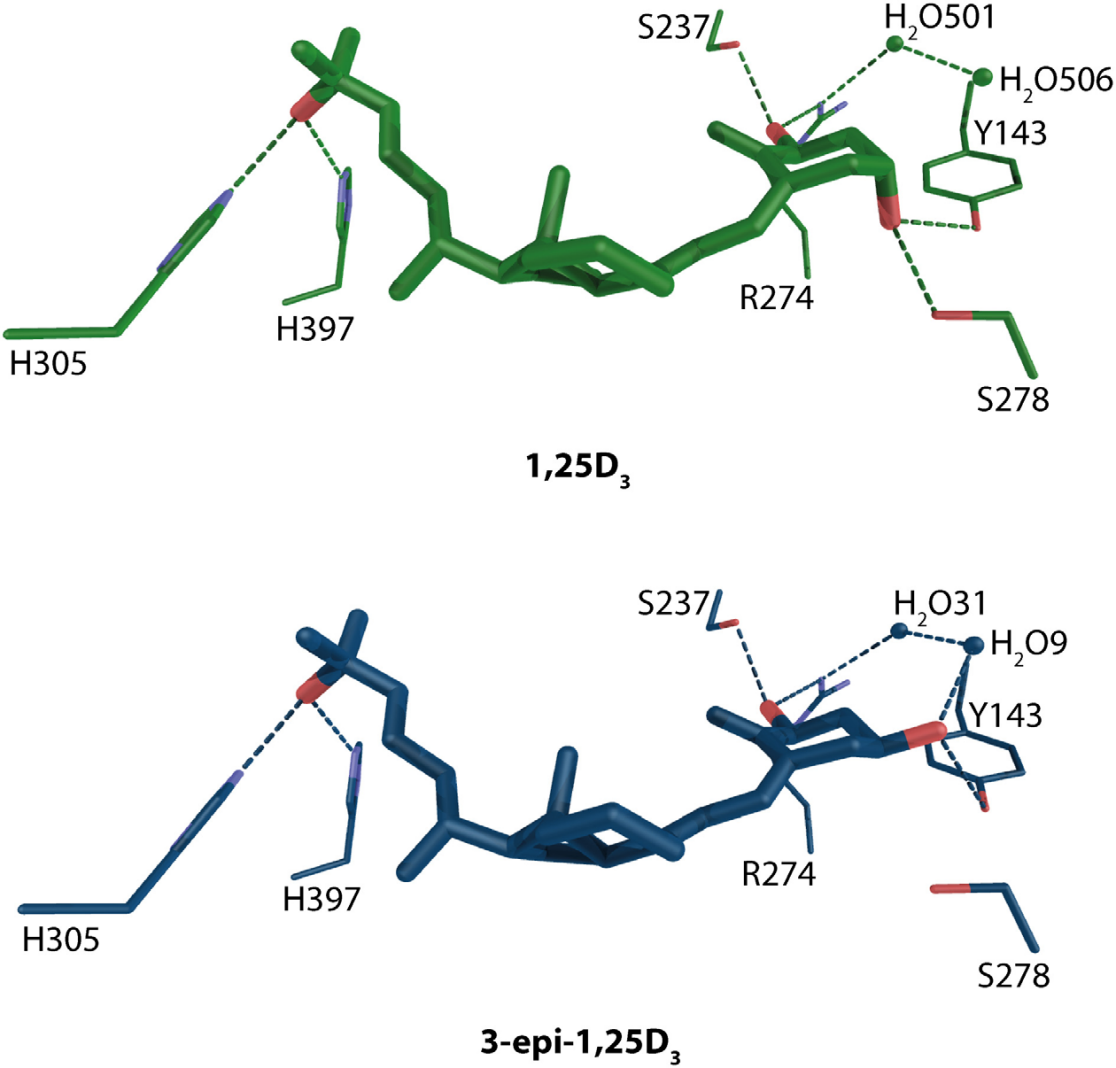
**Binding mode of 1α, 25-dihydroxyvitamin D_3_ and 3-epi-1α,25-dihydroxyvitamin D_3_ in the LBP of human VDR (source PDBID: 1DB1 and 3A78)**. 1,25D_3_ and 3-epi-1,25D_3_ are highlighted in green and blue color, respectively. The hydrogen bonds are in green and blue dashed lines.

Due to the space limitation this review cannot address and discuss all the VDR-ligand complexes, for some more details see text below or recent reviews (Carlberg and Molnár, 2012; Carlberg et al., 2012), but some of the structures with natural ligands will be discussed in more details. One of them is 3-epi-1α,25-dihydroxyvitamin D_3_ (3-epi-1,25D_3_) a 1,25D_3_ metabolite that has been shown to exhibit tissue specific activities comparable to 1,25D_3_ (Norman et al., 1993; Reddy et al., 2000). The structural analysis showed a binding mode very similar to that of 1,25D_3_ with interesting compensation for the lacking S278-3α-OH hydrogen bond for the epimer using water mediated contacts Figure 4 (Molnár et al., 2011). Interestingly, the same water channel is present in the 1,25D_3_ complex and was observed with other complexes as well (Tocchini-Valentini et al., 2001; Hourai et al., 2006).

**Figure 5:**
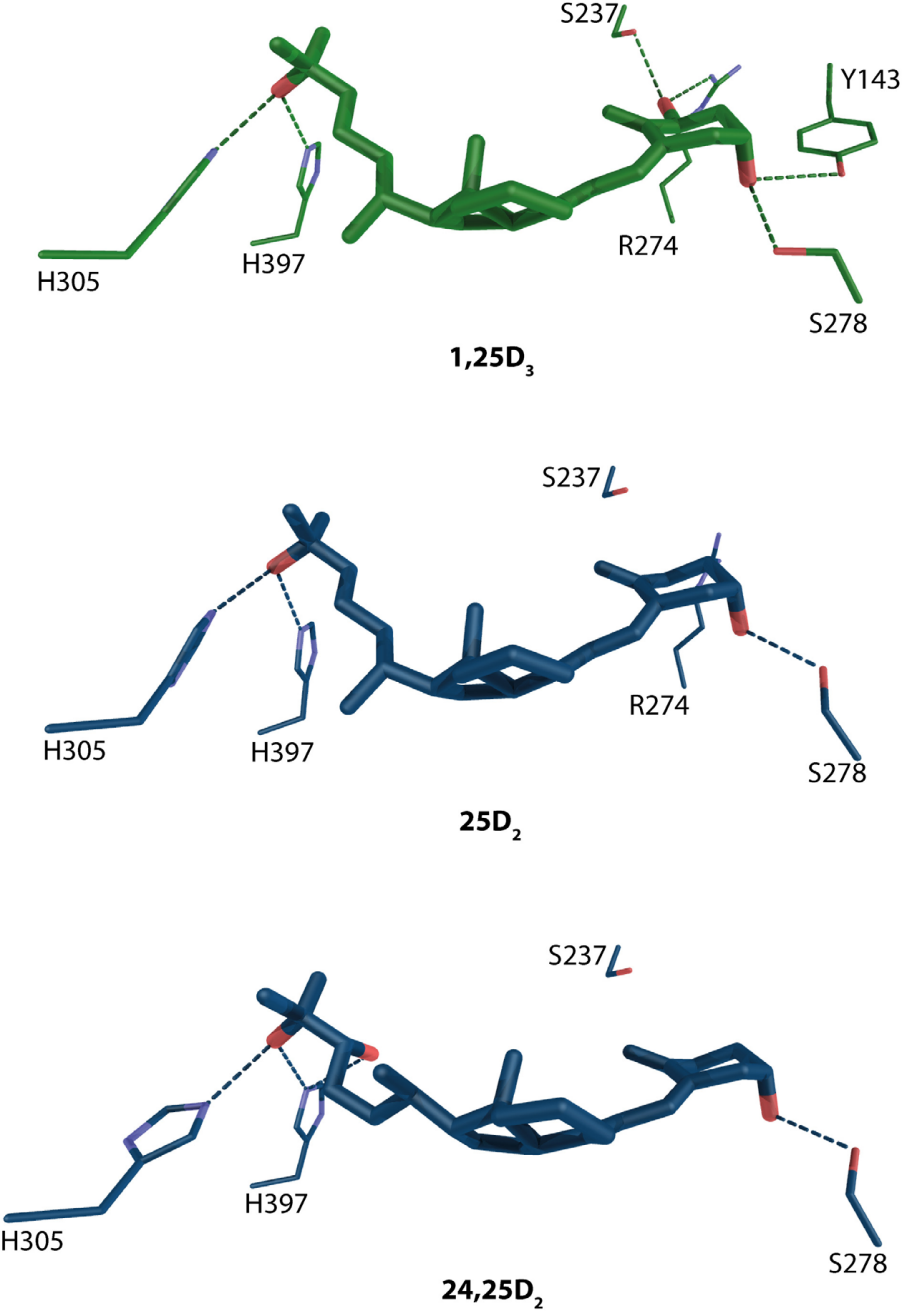
**Binding mode of 1α, 25-dihydroxyvitamin D_3_, 25-hydroxyvitamin D_3_ and its metabolite 24R,25-hydroxyvitamin D_3_ in the LBP of human VDR (source MD simulation Lou et al., 2010)**. 1,25D_3_ and 25D_3_/24,25D_3_ and are highlighted in green and blue color, respectively. The hydrogen bonds are in green and blue dashed lines.

A widely accepted fact is that precursor 25-hydroxyvitamin D_3_ (25D_3_) and its metabolite 24R,25-hydroxyvitamin D_3_ (24,25D_3_) does not posses significant biological activities. However, it has been shown that in *Cyp27b1*^−/−^ cells, that are unable to produce actively 1,25D_3_, the VD signaling may be primary mediated via 25D_3_ (Lou et al., 2010). It stays a matter of discussion that under physiological condition how much of the VD signaling is mediated via 1,25D_3_ vs. 25D_3_. 24,25D_3_ shows only weak potency of influencing VD signaling at concentration 500nM although an enhancement for human osteoblast differentiation has been shown at concentration 1 μM (van Driel et al., 2006). Docking and subsequent MD simulations have been done to see the binding mode of these compounds and it has been confirmed that the position of residues mediating the anchoring hydrogen bonds are conserved with the exception of R274 which is located further than 3.5 Å from 24,25D_3_ Figure 5. Another residue that is unable to make a binding contribution is S237, but its position in the pocket is maintained. The void created by the missing R274 increases the LBP and lowers the occupancy factor for this compound. The analysis of the simple binding mode confers the activity range from 1,25D_3_ > 25D_3_ > 24,25D_3_. This also shows in general the importance of the 1α-OH group for the potency of the VDR agonist.

**Figure 6:**
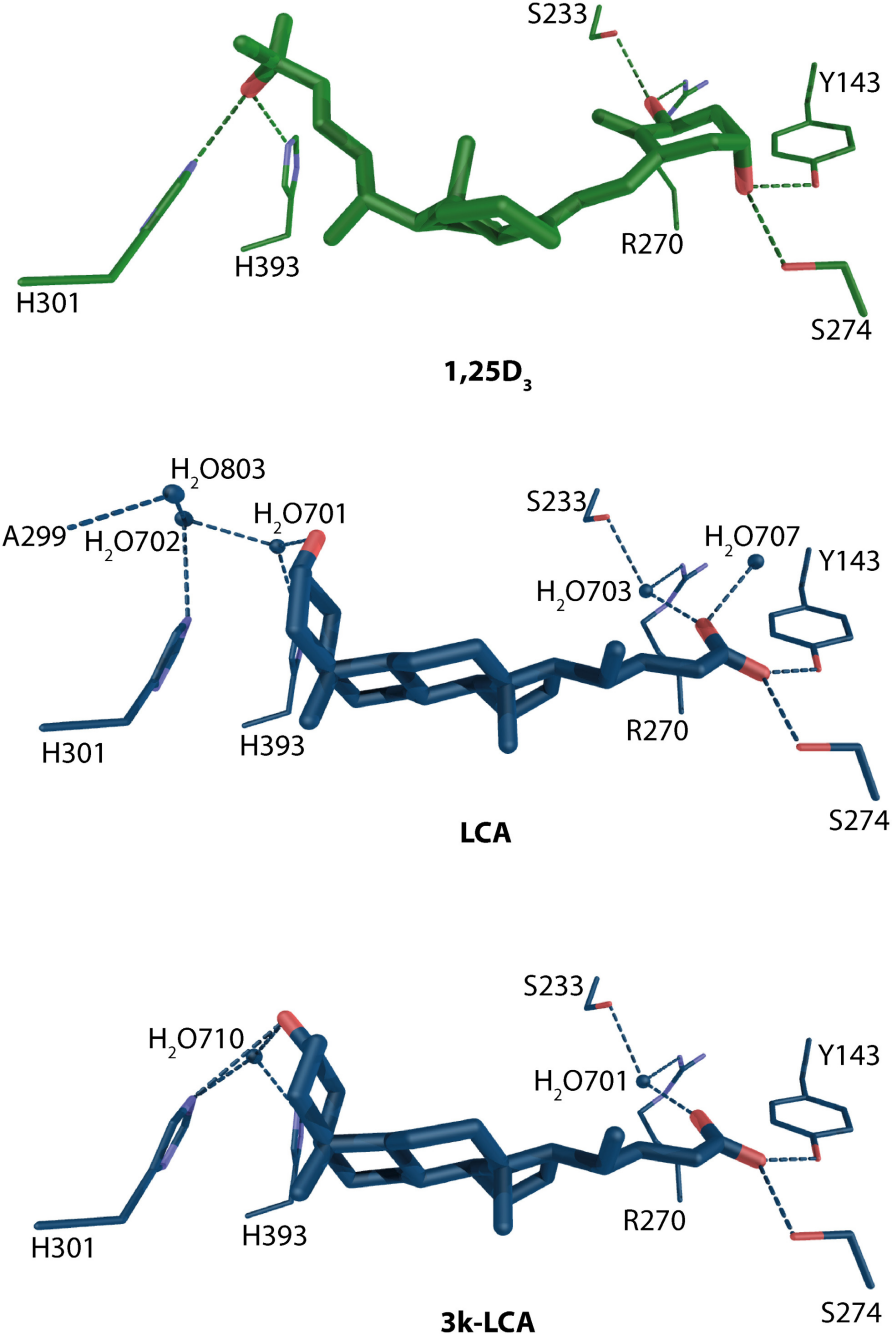
**Binding mode of 1α, 25-dihydroxyvitamin D_3_, lithocholic acid and 3-ketolitocholic acid in the LBP of rat VDR (source PDBID: 1RK3, 3W5P, and 3W5Q)**. 1,25D_3_ and LCA/3kLCA and are highlighted in green and blue color, respectively. The hydrogen bonds are in green and blue dashed lines.

Recently, the crystal structures for another group of natural ligands have been solved. From the identification of secondary bile acids as VDR agonist (Makishima et al., 2002) the interesting question remained how these compounds bind to VDR. From the structural data it is evident that the litocholic acid (LCA) and 3-keto litocholic acid (3kLCA) are located in the opposite orientation than 1,25D_3_ (Figure 6). The 24-carboxyl group faces the β-turns of VDR, the β-region of the steroid backbone the helix 6–7/11 region and the A-ring is in the direction of helix 12. The S274 (hVDR S278) and Y143 hydrogen bonds are conserved in all structures (Figure 6). The difference for this part of the ligand-binding comes from the water mediated contacts with both R270 (hVDR R274) and S233 (hVDR S237). These contacts seem to be weaker compare to 1,25D_3_ but not so weak as in case of 25D_3_ and 24,25D_3_, where in fact they are missing due to lack of 1α-OH group. The opposite part of the bile acids shows also weaker hydrogen bonding network than for the previously discussed VD metabolites including 1,25D_3_. The structural comparison between the two bile acids shows a less complex, more straightforward binding mode for 3kLCA with only one water molecule involved. Whereas, for LCA there are no direct contacts with H301 (hVDR H305) and H393 (hVDR H397). All these contacts are mediated thorough waters. This also may explain the lower VDR activation potential of 3kLCA compared to LCA.

I would seems that the evolutionary design of VDR reached by recognizing 1,25D_3_ its perfection. All the anchoring points show effective hydrogen bonding and by looking at the limited data for natural ligands any deviation from 1,25D_3_ will result in the use of alternative bridging contacts such water molecules. This shows a rather limited adaptability of VDR in effectively recognizing its natural ligands, yet larger than we envisioned a decade ago. One must keep in mind that the activation of VDR with one or another metabolite will largely depend on the local cellular concentration of this compounds leading for instance for favored activation via bile acids instead of classical 1,25D_3_ binding.

## DNA-recognition and binding

**Figure 7:**
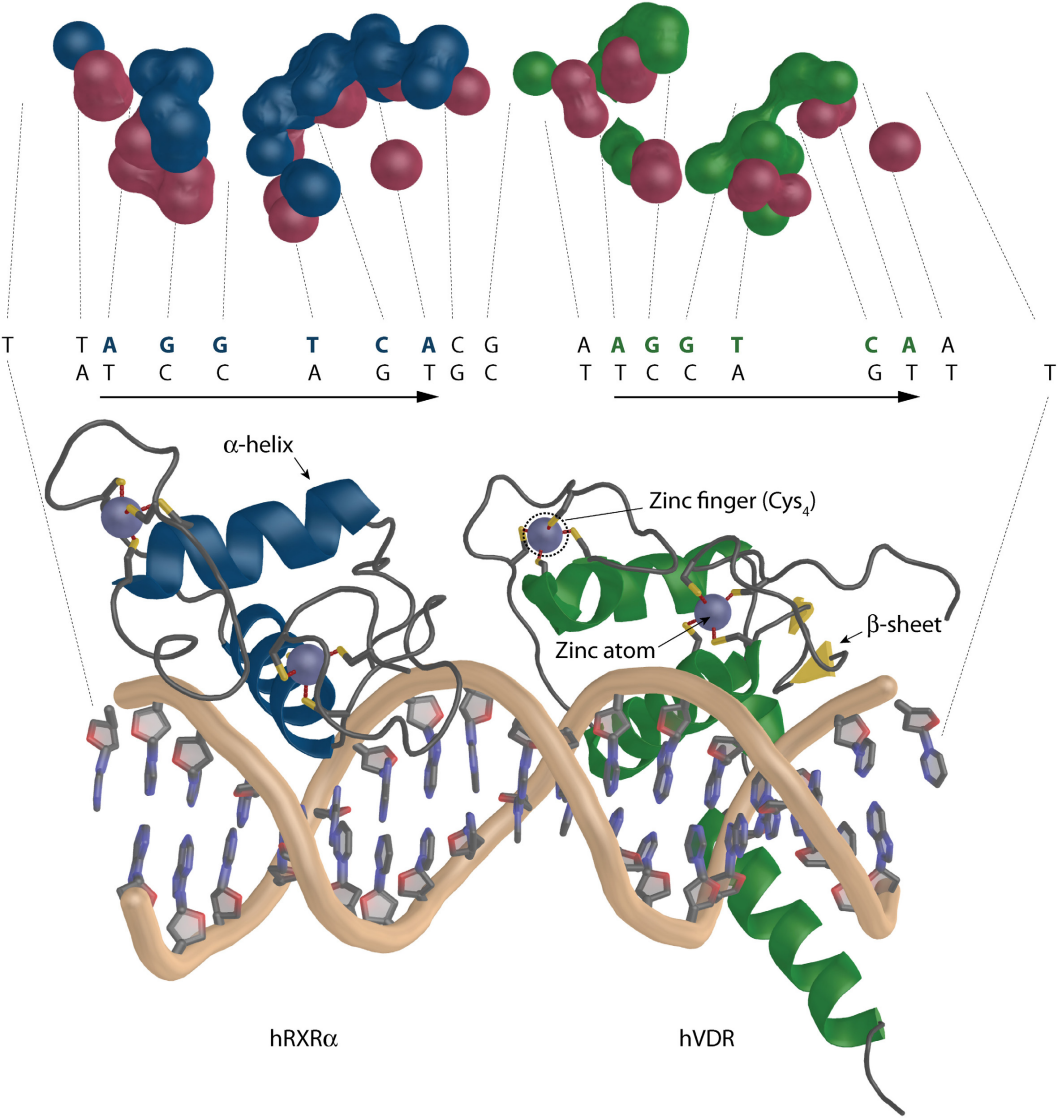
**The overall architecture of the DBD complex of RXR-VDR on canonical DR3 element (PDBID: 1YNW)**. The two zinc atoms (light blue spheres) with the respective cysteins are shown (bottom). RXR is shown in blue and VDR in green. The coiled protein regions are in gray and β-sheets in yellow. The surface representation of the contact atoms interacting between DNA and the heterodimer is shown (top). The proteins and DNA are visualized in different color DNA (red), RXR (blue), and VDR (green).

VDR belongs to the class of zinc finger transcriptional factors with DBD that consists of a highly conserved 66 aa residue core (Khorasanizadeh and Rastinejad, 2001) and an adjacent C-terminal extension. The conserved core has two zinc fingers where one contains fours cystein residues per atom of zinc (Figure 7). This feature allows VDR to effectively recognize and bind hormone response elements (REs) - termed VDREs. VDREs are typically made up of two hexameric half-sites whose consensus sequence is 5′-RGKTCA-3′, where R = A or G and K = G or T. The half-sites may be arranged in various orientation most commonly forming a direct repeat with three neutral base pairs separating the half-sites (DR3) (Umesono et al., 1991). The unliganded VDR can occupy its REs also as a homodimer (Carlberg et al., 1993). Upon binding of ligand, VDR interacts with RXR and forms a heterodimeric complex that binds to VDREs with 5′-prime bound RXR.

The data for structural view on VDR-DNA recognition is scarce. To date we have only four structures published where three represent VDR homodimers on direct repeat 3 (DR3) from mouse osteopontin (mSPP) (CAC*GGTTCA*CGA*GGTTCA*), rat osteocalcin promoter (rOC)(CAC*GGGTGA*ATG*AGGACA*) and a canonical DR3 element (cDR3) (CAC*AGGTCA*CGA*AGGTCA*) (Table 1) (Shaffer and Gewirth, 2002). The last structure represents the DBDs of the heterodimeric RXR-VDR on canonical DR3 element (cDR3) (TT*AGGTCA*CGA*AGGTCA*A) (Table 1) (Shaffer and Gewirth, 2004). The 66 to 70 aa of the DBD are structurally coordinated by two zinc atoms that create a structure (Figure 7), in which one short α-helix is interacting directly with the major groove of the DNA (Härd et al., 1990; Lee et al., 1993). The VDR homodimers show asymmetric head to tail arrangements. The experimentally determined range of affinities of DR3s used for crystallographic studies are mSPP > cDR3 > rOC with mSPP supporting both VDR homodimer and RXR-VDR heterodimer binding, cDR3 requiring 10x higher VDR homodimer protein levels and weak heterodimer binding, whereas rOC is unable to bind VDR homodimers and has a very weak heterodimer binding ability (Freedman and Towers, 1991; Nishikawa et al., 1993; Toell et al., 2000). The change at third position, a purine to pyrimidine, of the consensus half sites AG*G*TCA allows the additional water mediated hydrogen contact of E42 with the DNA, which increase the stability of the mSPP-VDR complex. In case of rOC the reason for diminished VDR homodimer binding is because there is a G at position five of the upstream half-site GGGT*G*A, where supposedly RXR is bound in case of heterodimer binding. In high affinity half-sites in this location there is a C in the first strand but a complementary G in the second DNA strand. The interaction, which involves hydrogen bonds is between the R50 of VDR and the G from the second complementary strand. In rOC upstream half-site instead of G there is a C in the second strand, which is not a hydrogen bond acceptor thus R50 cannot interact with it. There is also some agreement between the strength of the homodimer binding and a sum of all existing DNA-VDR contacts within 3.5 Å calculated with the *ncont* program of the CCP4 suite (Winn et al., 2011). The results show 88, 85, and 83 contacts for rOC, cDR3, and mSPP, respectively. Interesting is also the interacting surface ratio between the two VDR homodimer molecules in percentage (5′upstream: 3′downstream) 38.52:61.48% for rOC, 46.21: 53.8% for the cDR3 and a reversed ratio of 57.3:42.7% for mSPP. This would suggest that for strong VDR homodimer binding there is a more contribution from the upstream half-site, but a clear conclusion cannot be reached based on this limited data set. In the RXR-VDR crystal structure there are two asymmetric units where the full complex shows an orientation RXR to VDR for unit 2 and a reversed orientation VDR to RXR for unit 1 with RXR bound on the downstream part of the VDRE. This may be due to the stabilization contacts between the adjacent VDR molecules where the hinge of one VDR molecule is stabilized with the DBD of the second VDR molecule. The general organization of the unit 2 is depicted also on the Figure 7 bottom. The two zinc atoms (light blue color) with the respective cysteins are visualized (the second heterodimer unit is missing from the representation). The overall number of contacts is only 65, which is much lower than in case of VDR homodimers discussed earlier. This indicates that the binding of the RXR-DBD heterodimer to cDR3s is not optimal and for an effective binding a certain point of VDRE degeneration is needed. Moreover, the contribution of the monomers to the binding is rather interesting. The ratio between interacting monomeric surfaces is 56.22:44.26% (RXR-VDR) suggesting a higher contribution of RXR to the binding Figure 7 top (see also the H/D exchange experiments discussed below). This is most likely due to the reverse orientation of the RXR-VDR on the cDR3. The surfaces of the interacting atoms are visualized in different color DNA (red), RXR (blue) and VDR (green). It is to be noted that compared to DNA-protein interaction there are hardly any interactions between the protein monomers. None for RXR-VDR, for VDR homodimers there are two for mSPP and rOC, and five on cDR3. This is quite in agreement with full length receptor studies which suggest that most of the heterodimerisation is contributed from hinges and LBDs.

## The outside shell: partnering and the complex view

### VDR interacting protein partners

For VDR to function effectively as a regulator of transcription it is inevitable to interact with various protein partners. They show high structural and functional diversities ranging from enzymes, co-integrators and cofactors to components of distinct transduction signaling pathways. A comprehensive list of these partners with the accompanying citations are listed in Table 2.

**Table 2:** **List of VDR interacting proteins**.

**Name**	**Gene symbol/alternative name**	**Role**	**References**
Alien	ALIEN	Transcriptional corepressor	Polly et al., 2000
Androgen receptor-associated protein 54	ARA54^*^	Transcriptional coactivator	Ting et al., 2005
Androgen receptor-associated protein 70	ARA70	Transcriptional coactivator implicated in cancer	Ting et al., 2005
Brahma-related gene 1	BRG1/SMARCA4	ATPase subunit of the SWI/SNF complex	Fujiki et al., 2005
CREB-binding protein	CBP	Transcriptional cointegrator	Castillo et al., 1999
Cyclin D3	CCD3	Subunits of the cyclin-dependent kinases	Jian et al., 2005
Cyclin-dependent kinase 7	CDK7/hMo15	Component of the TFIIH transcription complex	Nevado et al., 2004
CXXC finger 5	CXXC5	Cell cycle regulation	Marshall et al., 2012
E1A binding protein p300	p300	Transcriptional cointegrator	Kim et al., 2005
Fas-activated serine/threonine kinase	FASTK	Involvement in splicing	Marshall et al., 2012
Feline Gardner-Rasheed sarcoma viral oncogene homolog	FRG	Signal transduction (protein tyrosine kinase)	Ellison et al., 2005
General transcription factor IIB	TFIIB	Subunit of the basal transcription machinery	Nevado et al., 2004
Hairless	HR	Transcriptional corepressor	Hsieh et al., 2003
High mobility group nucleosomal binding domain 3	HMGN3/TRIP7	Possible chromatin modifier	Albers et al., 2005
Histone deacetylase 2	HDAC2	Histone modifier	Fujiki et al., 2005
Ligand-dependent NR corepressor	LCOR	Transcriptional corepressor	Fernandes et al., 2003
Mediator complex subunit 1	MED1/TRAP220/RIP205/PPARBP	Transcriptional regulation/part of the mediator	Rachez et al., 1999
Mediator complex subunit 4	MED4/DRIP36/p34	Transcriptional regulation/part of the mediator	Rachez et al., 1999
Mediator complex subunit 6	MED6/DRIP33	Transcriptional regulation/part of the mediator	Rachez et al., 1999
Mediator complex subunit 7	MED7/DRIP34	Transcriptional regulation/part of the mediator	Rachez et al., 1999
Mediator complex subunit 12	MED12/DRIP240/ARC240/TRAP230	Transcriptional regulation/part of the mediator	Rachez et al., 1999
Mediator complex subunit 16	MED16/DRIP92/TRAP95	Transcriptional regulation/part of the mediator	Rachez et al., 1999
Mediator complex subunit 17	MED16/DRIP77/TRAP80	Transcriptional regulation/part of the mediator	Rachez et al., 1999
Mediator complex subunit 23	MED23/DRIP130/CRSP130	Transcriptional regulation/part of the mediator	Rachez et al., 1999
Mothers against decapentaplegic homolog 3	SMAD3	Transcriptional coactivator	Yanagisawa et al., 1999
Myosin light chain 3	MYL3	Regulatory light chain of myosin	Marshall et al., 2012
NR coactivator 1	NCOA1/SRC1	p160 family coactivator	Castillo et al., 1999
NR coactivator 2	NCOA2/TIF2/GRIP1	p160 family coactivator	Hong et al., 1997)
NR coactivator 3	NCOA3/RAC3/SRC3/AIB1	p160 family coactivator	Molnár et al., 2005
NR coactivator 6	NCOA6/PRIP/ASC2	Transcriptional coactivator	Mahajan and Samuels, 2000
NR corepressor 1	NCOR1	Transcriptional corepressor	Tagami et al., 1998
NR corepressor 2	NCOR2/SMRT/TRAC2	Transcriptional corepressor	Kim et al., 2009
NR subfamily 0, group B, member 2	NR0B2 (SHP)	Negative transcriptional regulator	Albers et al., 2005
NR subfamily 4, group A, member 1	NR4A1 (NGFIB)	Expression genes during liver regeneration	Marshall et al., 2012
p53	PT53	Tumor supression	Stambolsky et al., 2010
Receptor-associated protein 46	RAP46/BAG1	Regulation of cell growth in response to stress	Guzey et al., 2000
Receptor-interacting protein 140	RIP140/NRIP1	Coregulator with selective properties	Albers et al., 2005
Retinoblastoma 1	RB	NR coregulator/tumor suppressor	Chan and Hong, 2001
Retinoblastoma-binding protein 2	RBP2	Transcriptional coactivator	Chan and Hong, 2001
Retinoid X receptor α	RXRα	Heterodimeric VDR partner	Liu et al., 2000
Retinoid X receptor β	RXRβ	Heterodimeric VDR partner	Rachez et al., 1999
Retinoid X receptor γ	RXRγ	Heterodimeric VDR partner	Albers et al., 2005
Protooncogene c jun	JUN	Transcriptional factor	Towers et al., 1999
SIN3 homolog A, transcriptional regulator (yeast)	SIN3A	Transcriptional corepressor/cointergrator	Fujiki et al., 2005
SKI interacting protein	SKIP/SNW1/NCoA-62	Transcriptional coactivator	Baudino et al., 1998
Suppressor of RNA polymerase B 7	SRB7	Transcriptional coactivator	Ito et al., 1999
Thymine-DNA glycosylase	TDG	Coregulator/base excision repair	Chen et al., 2003
Thyroid receptor-interacting protein 1	TRIP1/SUG1/PSMC5	CAD (Conserved ATPase domain) protein	Masuyama and Hiramatsu, 2004
Transcriptional intermediary factor 1	TIF1α/CCCP	Coregulator with selective properties	Thénot et al., 1997
Tropomyosin	TPM2	Possible role in receptor Internalization	Marshall et al., 2012
Vitamin D receptor-interacting protein (100kD)	DRIP100/ARC100/TRAP100	VDR associated DRIP complex	Rachez et al., 1999
Vitamin D receptor-interacting protein (150kD)	DRIP150/ARC150/TRAP170	VDR associated DRIP complex	Rachez et al., 1999
Vitamin D receptor-interacting repressor	VDIR/TCF3/ITF1	Negative regulator of the CYP27B1	Kim et al., 2007
Williams syndrome transcription factor	WSTF/BAZ1B	Recruitment of unliganded VDR to target promoters	Fujiki et al., 2005
Xin actin-binding repeat containing protein 1	XIRP1	Protects actin filaments from depolymerization	Marshall et al., 2012

* no direct physical interaction but has positive effect on VDR transactivation.

One of the first complex identified using co-immuno-precipitation from mammalian cells was the VDR interacting protein DRIP complex, which is recruited to VDR in a completely ligand-dependent manner (Rachez et al., 1999). Many of the its components were shared with the earlier identified thyroid hormone receptor (TR) interacting protein complex TRAP (Fondell et al., 1996). It has been no surprise that majority of the interacting proteins can be related directly to transcriptional regulation such as subunits of the mediator complex MED1, 4, 6, 7, 12, 16, 17, and 23 (Rachez et al., 1999) or cofactors such as coactivators NCOA1-3 (Hong et al., 1997; Castillo et al., 1999; Molnár et al., 2005), NCOA6 (Mahajan and Samuels, 2000), ARA54 (Ting et al., 2005), SKIP (Baudino et al., 1998), RBP2 (Chan and Hong, 2001), SRB7 (Ito et al., 1999) and corepressors Alien (Polly et al., 2000), NCOR1 and 2 (Tagami et al., 1998; Kim et al., 2009), SIN3A (Fujiki et al., 2005), LCOR (Fernandes et al., 2003). Others show more selective properties functioning as coactivators and corepressors depending on the particular conditions such as RIP140 (Albers et al., 2005) or TIF1α (Thénot et al., 1997). Some are implicated in cellular processes such as cell cycle regulation CDK7 (Nevado et al., 2004), RAP46 (Guzey et al., 2000), DNA repair TDG (Chen et al., 2003) or signaling cascade FRG (Ellison et al., 2005). Interesting is the interaction and thus possible crosstalk with other NRs such as SHP (Albers et al., 2005), which lacks DBD and has corepressor-like behavior, and NGFIB, which has been shown to have a role during liver regeneration (Marshall et al., 2012). Some of the newly identified protein partners that may implicate VDR's involvements in new processes are XIRP1 that protects actin filaments from depolymerization or MYL3 a regulatory light chain of myosin (Marshall et al., 2012).

### Structural data of SRC1 and MED1/DRIP205 interaction with VDR

**Figure 8:**
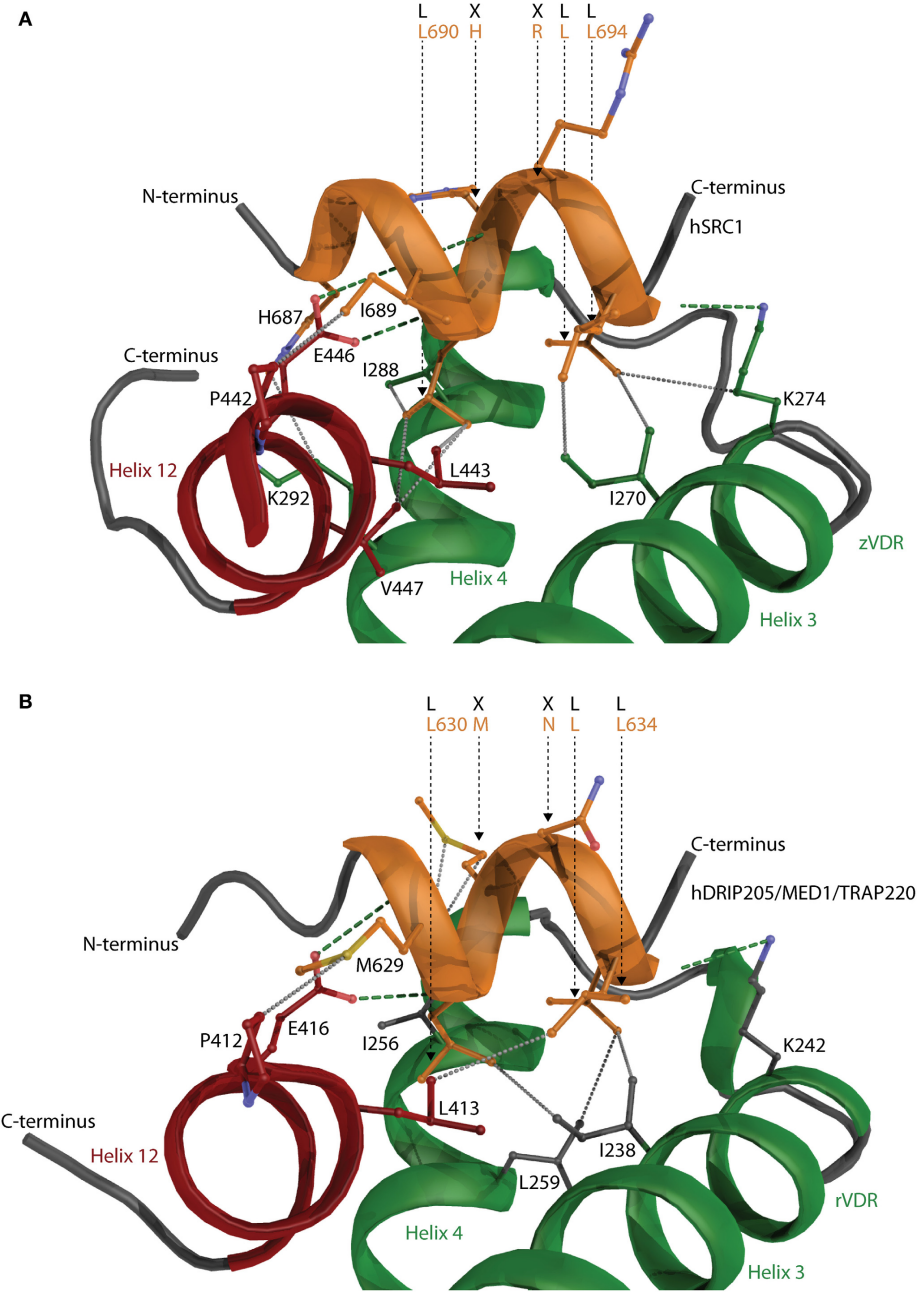
**The interaction of coactivator peptides with VDR**. Peptides derived from **(A)** steroid receptor coactivator 1 (SRC1) with zVDR and **(B)** mediator complex subunit 1 (MED1/DRIP205) with rVDR shown. SRC1 and MED1 is shown in orange and VDR in green. Helix 12 is highlighted in red. The hydrogen bonds and hydrophobic interactions are visualized with green and gray dashed lines, respectively. The important residues such as the conserved “charge clamp” glutamate from helix 12 and lysine in helix 3 contributing to the CoA-VDR interaction are also shown.

There is a big limitation in obtaining large transcriptional complexes, which is firstly due to the transient nature of the complex where VDR serves as a docking and acquiring platform bringing other proteins that either act as chromatin modifiers, parts of the mediator, of various cofactors and bridging factors to the close proximity of the functional VDREs. The complex may be assembled for a short moment to initiate and/or repress the transcription thereafter fulfilling this action it falls apart. The second reason might be that many of the interacting proteins such as cofactors show a high degree of disorder. The crystallization of unfolded proteins is very tricky and many times even impossible. The intrinsic disorder of a VDR interacting proteins is an expected structural property since e.g., members of the p160 general coactivator family have to adopt and interact with many various transcription factors. Thus structural data from only short interacting peptides derived from steroid receptor coactivator 1 (SRC1) with zVDR (Figure 8A) and mediator complex subunit 1 (MED1/DRIP205) with rVDR are available showing only the core interaction between VDR and the LXXLL motif of coactivators (Figure 8B). Both peptides interact in a very similar fashion. The α-helix of the peptide is oriented with its N-terminus toward helix 12. The two peptides interact through their LXXLL motifs, LHRLL in SRC1 and LMNLL in MED1, and most of the interaction is contributed from hydrophobic contacts of coactivator's leucine residues with the hydrophobic core from VDR helices 3, 4, and 12. The anchoring points of the short α-helix are based on the interaction with the “charge clamp” consisting of the conserved glutamate in helix 12 and lysine in helix 3, and the backbone amides of the coactivator peptide. The similar interaction of the two LXXLL motifs raises the question on how the specificity is achieved in the interaction. The situation is complicated with the fact that some of the coactivators have more than one interaction motif such as SRC1 has five of them that are similar or related to LXXLL motifs, but so far only three of them were reported/studied in detail.

### SAXS, cryo-EM, and H/D exchange studies with full length RXR-VDR complexes

**Figure 9:**
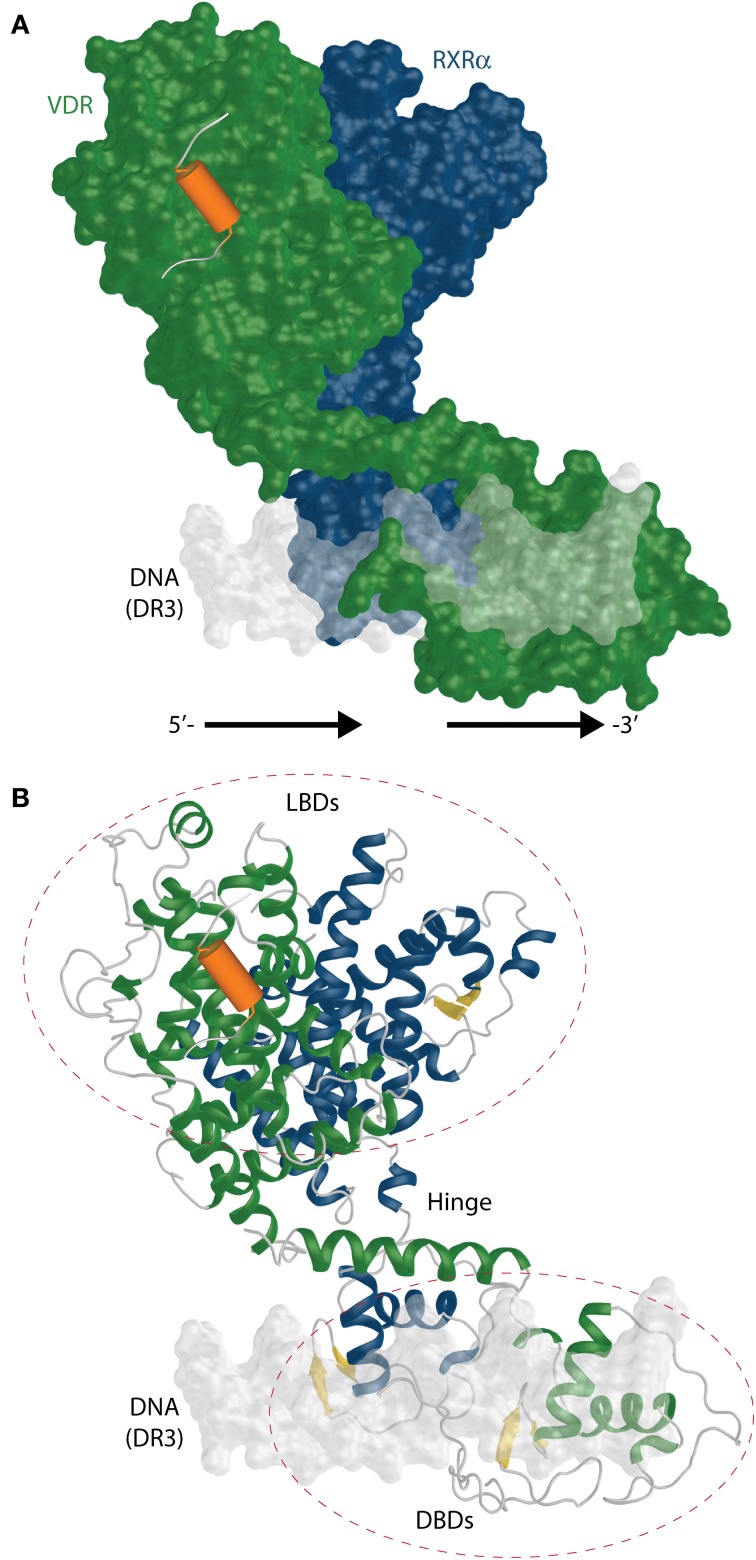
**The full length RXR-VDR structural model derived from SAXS and cryo-EM experiments. (A)** A surface representation of the RXR(blue)-VDR(green) heterodimer is shown on DR3 VDRE. The possible location of the coactivator peptide (orange) is highlighted as well. The 5′- and 3′-prime orientation of the DR3 is annotated. **(B)** Ribbon representation of the same complex shows the relative organization and fold of LBD, DBD and the connective hinge between them. The β-sheets are shown in yellow color.

The recent studies with VDR complexes in solution that employed the use of modern techniques such as SAXS, cryo-EM, HDX with full length VDR-RXRα showed somewhat more comprehensive perspective for the 3D organization and possible function of the VDR-RXRα-cofactor complex. The SAXS (Rochel et al., 2011) and cryo-EM (Orlov et al., 2012) derived model is shown in Figure 9A.

Upon binding to DNA from osteocalcin VDRE, the SAXS data derived RXR-VDR shows an elongated asymmetric open conformation with separate DBDs and LBDs and a well structured VDR hinge with VDR located downstream and RXR on upstream half sites (Rochel et al., 2011) (Figure 9B). On contrary the coiled structure of the RXR hinge allows its adaptability to different REs. The hinges play an important role in an open conformation. The hinges also underly one of the very important feature of the RXR-VDR complex, the dynamic character. The DBD binding to DR3 results in a rotation of the LBD dimers take around 90° with respect to the DNA (Figure 9A). The same study showed that coactivators MED1/DRIP205 and SRC1 have higher affinity to VDR compared to RXR. This points to the binding of only one molecule of coactivator through VDR, which is not supporting the “deck model” of binding (one LXXLL motif to VDR and the other to RXR) for these coactivators. Studies using mutants show the preferential binding of VDR to the second LXXLL motif of MED1/DRIP205 compared the weak recruitment of the first motif to RXR (Ren et al., 2000). However, both motifs are crucial for the effectivity of the NR activation complex *in vivo* (Malik et al., 2004). RXR may play a role in coactivator recruitment as well by associating to some other factors.

The cryo-EM studies of the RXR-VDR have a higher resolution than SAXS data and it is possible to obtain more precise electron densities for VDR A/B and hinge domains for both receptors (Orlov et al., 2012). The heterodimer takes an L-shape form on the DR3 with a proper orientation of RXR on the upstream and VDR on the downstream half site (similar to SAXS model). The complex supports also the asymmetric open architecture from SAXS data. Both LBDs are positioned above the 5′ upstream half site as shown on Figure 9A. This result also emphasizes the importance of the hinges for the correct function of the complex. The main nature of the flexible hinge of RXR allows to contact the CTE helix with helix 1 and makes it possible to adopt differential spacing in DR REs. In addition, the coiled RXR hinge has to be long to reach DBD to its LBD which is located on the opposite side of the DR3 VDRE. The cryo-EM data points to one interesting feature, the potential in modulation of the DNA-binding using the 17 aa long A/B domain of VDR, which in fact interacts with the major groove of the DNA.

The H/D exchange (HDX) is a great tools to address the dynamic properties of the RXR-VDR-SRC1 complex (Zhang et al., 2011). The addition of RXR to VDR stabilizes region with helices 6–7, very similar to what is seen when some of agonists bind to VDR. As expected, upon 1,25D_3_ binding in the VDR LBD the helices 1, 3, 5–7, and 11 (the actual region that forms the LBP) have been stabilized, but binding efficiency of RXR to VDR is not enhanced. For RXR the helices 7 and 10 are stabilized (increase of the heterodimerisation) and an allosteric communication has been shown for the helix 3 of RXR. The 9-*cis* RA binding in general stabilized RXR, but in contrast to the crystal structure, it increased the fluctuation in helix 12. This observation may also indicate that the crystal structure take the minimized energy conformation of the complex, but in reality it is more dynamic. The allosteric communication in VDR upon 9-*cis* RA binding was seen in the helices 3, 5, and 7 that are adjacent from the heterodimerisation interface pointing to modulation of the complex upon only 9-*cis* RA binding. Surprisingly, the concurrent binding of 1,25D_3_ and 9-*cis* RA has a destabilizing affect on the VDR DBD. A stronger increase of the VDR binding has been observed compared to RXR DBD in the presence of cDR3 element and in the absence of ligands, pointing to the higher contribution of the VDR for DNA interaction. In addition, interesting allosteric stabilization were observed for the VDR hinge and for helices 7–8 and 9–10. Moreover, unexpectedly the helix 12 of VDR showed increased fluctuation. However, it should be noted that in this experimental setup a coactivator has been missing from the protein complex (see below). The binding to the natural VDRE from *CYP24A1* gene showed similar result except it seemed that 5′-AGGTCA-3′ half-site was occupied by VDR and helix 12 of RXR was more stable. Unexpectedly, the stability of the heterodimer on *CYP24A1* VDRE was reduced although the binding affinities of the two VDREs are in the same magnitude. The interaction of the coactivator SRC1, that contained three LXXLL motifs, with the heterodimer bound to both 1,25D_3_ and 9-*cis* RA increased the stability of VDR's helix 12 and helices 3 and 10–11 of RXR. Helices 3 and 4 of VDR cannot be further stabilized since they achieve maximal stabilization upon 1,25D_3_ binding. For RXR the loop between helices 10 and 11 is important in the formation of the hydrophobic groove facilitating coactivator binding. Besides the classical charge clamp RXR contains the so called “aromatic clamp” consisting of residues in helices 3 (F437, F277) and 12 (F450) that is important for coactivator binding. As expected in the absence of both ligand no coactivator interaction was observed and the separate addition of the 1,25D_3_ or 9-*cis* RA recruited the coactivator in a ligand-specific manner. Further HDX and cell based experiments showed that a simultaneous binding of the coactivator to both receptors is important and in the interaction with the RXR-VDR heterodimer only one SRC1 molecule is required (Zhang et al., 2011). This is in contrast to the SAXS derived model (Rochel et al., 2011). For the RXR-VDR-SRC1 complex the helix 12 of VDR has been stabilized upon addition of the DNA. In addition, HDX shows that the DNA-binding enhances the recruitment of SRC1 to RXR, thus the binding of the DNA stabilizes the recruitment of SRC1 to the whole heterodimer not just to the partner such as VDR.

Both, the SAXS and cryo-EM, studies highlighted the open architecture conformation in solution unlike it has been shown for the full length PPAR-RXR (Chandra et al., 2008). The recent crystal structure of the full length RXRα–LXRβ on DR4 RE provides also a support for the open conformation of the NR heterodimer complex (Lou et al., 2014). It will have to be seen whether the closed PPAR-RXR complex on DR1 is and exception, although more plausible is the open conformation giving a rather high dynamic freedom for the NR heterodimer in the large transcriptional complex.

## Conclusions

The main aim of this review was to collect and discuss structural data that is related to vitamin D signaling. The structural data for various isolated domains (LBD and DBD) show their organization on atomic level. This data is sufficient for understanding the particular ligand- and DNA-protein interactions but fails to provide spatial information on the mutual orientation of the domains of RXR-VDR on its natural promoters. It also fails to highlight inter-domain communication after DNA-, ligand- or cofactor-binding. However, they are irreplaceable tools for structure-based drug design and mechanistic view of the VDR action. The dissemination of the information derived from structural data and *in silico* models may help to understand how VDR works in its natural settings and provides a space for the intervention in various diseases. The recent SAXS, Cryo-EM and H/D exchange studies with full length RXR-VDR complexes show more complex views on VDR function and provide first tools for the integration of structural information with genomic, epi-genomic, transcriptional and functional data.

## Future perspective

There are numerous scientific questions connected to VDR that may be interesting to answer on structural level. The list is not complete but here are some of them:

- Do the subtle differences between the various ligand-bound VDR complexes indeed represent the differences in the biological specificities and activities *in vivo*?
- Can the LBP related measurements and other descriptors be related to the potency of VDR ligands?
- Are there some additional natural compounds that are able to bind VDR and thus influence the metabolism and detoxification?
- What is the real role of the insertion domain in VDR?
- Is it possible to relate structurally the binding affinities of the RXR-VDR complexes with DNA?
- Are there other *in vivo* significant binding modes of VDR to the DNA such as monomers?
- What is the architecture of the RXR-VDR complex on non-DR3 VDREs and in indirect DNA interactions?
- How the recruitment specificity of the LXXLL containing proteins is regulated in the cell?
- How is the inter-domain communication precisely mediated upon ligand- DNA- and protein-VDR interaction?

### Conflict of interest statement

The author declares that the research was conducted in the absence of any commercial or financial relationships that could be construed as a potential conflict of interest. The Editor Carsten Carlberg declares that, despite being affiliated to the same institution as the author Ferdinand Molnár, the review process was handled objectively and no conflict of interest exists.

## Abbreviations

1,25D_3_: 1α, 25-dihydroxyvitamin D_3_
3D: three-dimensional
3Klca: 3-keto lithocholic acid
9-*cis* RA: 9-*cis* retinoic acid
aa: amino acids
cryo-EM: cryo-electron microscope
*Cyp27b1*: 25-hydroxyvitamin D_3_ 1α-hydroxylase; *D.*rerio, Danio rerio
DBD: DNA-binding domain
DNA: deoxyribonucleic acid
DR: direct repeat
DRIP: vitamin-D-receptor interacting protein
h: human
*H. sapiens*: *Homo sapiens;* HDX, H/D exchange
LBD: ligand-binding domain
LBP: ligand-binding pocket
LCA: lithocholic acid
MD: molecular dynamics
OH: hydroxyl
*P. marinus*: Petromyzon marinus
PXR: pregnane X recptor
RAR: Retinoic acid receptor
r: rat
*R. norvegicus*: *Rattus norvegicus;* RE, response element
RID: receptor interaction domain
RXR: retinoid X receptor
SAXS: short angle X-ray scattering
TR: thyroid hormone receptor
VD: vitamin D
VDR: vitamin D receptor
VDRE: VD response element
z: zebrafish.

## References

[B1] Albers M. Kranz H. Kober I. Kaiser C. Klink M. Suckow J. (2005). Automated yeast two-hybrid screening for nuclear receptor-interacting proteins. Mol. Cell. Proteomics 4, 205–213 10.1074/mcp.M400169-MCP200 15604093

[B2] Antony P. Sigüeiro R. Huet T. Sato Y. Ramalanjaona N. Rodrigues L. C. (2010). Structure-function relationships and crystal structures of the vitamin D receptor bound 2 α-methyl-(20S,23S)- and 2 α-methyl-(20S,23R)-epoxymethano-1 α,25-dihydroxyvitamin D_3_. J. Med. Chem. 53, 1159–1171 10.1021/jm9014636 20070104

[B3] Asano L. Ito I. Kuwabara N. Waku T. Yanagisawa J. Miyachi H. (2013). Structural basis for vitamin D receptor agonism by novel non-secosteroidal ligands. FEBS Lett. 587, 957–963 10.1016/j.febslet.2013.02.028 23462137

[B4] Baudino T. A. Kraichely D. M. Jefcoat S. C. Winchester S. K. Partridge N. C. MacDonald P. N. (1998). Isolation and characterization of a novel coactivator protein, NCoA-62, involved in vitamin D-mediated transcription. J. Biol. Chem. 273, 16434–16441 10.1074/jbc.273.26.16434 9632709

[B5] Bourguet W. Ruff M. Chambon P. Gronemeyer H. Moras D. (1995). Crystal structure of the ligand-binding domain of the human nuclear receptor RXRα. Nature 375, 377–82 10.1038/375377a0 7760929

[B6] Carlberg C. Bendik I. Wyss A. Meier E. Sturzenbecker L. J. Grippo J. F. (1993). Two nuclear signalling pathways for vitamin D. Nature 361, 657–660 10.1038/361657a0 8382345

[B7] Carlberg C. Molnár F. (2012). Current status of vitamin D signaling and its therapeutic applications. Curr. Top. Med. Chem. 12, 528–547 10.2174/156802612799436623 22242854

[B8] Carlberg C. Molnár F. Mouriño A. (2012). Vitamin D receptor ligands: the impact of crystal structures. Exp. Opin. Ther. Pat. 22, 417–435 10.1517/13543776.2012.673590 22449247

[B9] Castillo A. I. Jiménez-Lara A. M. Tolón R. M. Aranda A. (1999). Synergistic activation of the prolactin promoter by vitamin D receptor and GHF-1: role of the coactivators, CREB-binding protein and steroid hormone receptor coactivator-1 (SRC-1). Mol. Endocrinol. 13, 1141–1154 10.1210/mend.13.7.0320 10406465

[B10] Chan S. W. Hong W. (2001). Retinoblastoma-binding protein 2 (Rbp2) potentiates nuclear hormone receptor-mediated transcription. J. Biol. Chem. 276, 28402–28412 10.1074/jbc.M100313200 11358960

[B11] Chandra V. Huang P. Hamuro Y. Raghuram S. Wang Y. Burris T. (2008). Structure of the intact PPAR-γ-RXR-α nuclear receptor complex on DNA. Nature 456, 350–356 10.1038/nature07413 19043829 PMC2743566

[B12] Chen D. Lucey M. J. Phoenix F. Lopez-Garcia J. Hart S. M. Losson R. (2003). T:G mismatch-specific thymine-DNA glycosylase potentiates transcription of estrogen-regulated genes through direct interaction with estrogen receptor α. J. Biol. Chem. 278, 38586–38592 10.1074/jbc.M304286200 12874288

[B13] Ciesielski F. Rochel N. Moras D. (2007). Adaptability of the Vitamin D nuclear receptor to the synthetic ligand Gemini: remodelling the LBP with one side chain rotation. J. Steroid Biochem. Mol. Biol. 103, 235–242 10.1016/j.jsbmb.2006.12.003 17218092

[B14] Ciesielski F. Sato Y. Chebaro Y. Moras D. Dejaegere A. Rochel N. (2012). Structural basis for the accommodation of bis- and tris-aromatic derivatives in vitamin D nuclear receptor. J. Med. Chem. 55, 8440–8449 10.1021/jm300858s 22957834

[B15] Eelen G. Valle N. Sato Y. Rochel N. Verlinden L. de Clercq P. (2008). Superagonistic fluorinated vitamin D_3_ analogs stabilize helix 12 of the vitamin D receptor. Chem. Biol. 15, 1029–1034 10.1016/j.chembiol.2008.08.008 18940664

[B16] Eelen G. Verlinden L. Rochel N. Claessens F. de Clercq P. Vandewalle M. (2005). Superagonistic action of 14-epi-analogs of 1,25-dihydroxyvitamin D explained by vitamin D receptor-coactivator interaction. Mol. Pharmacol. 67, 1566–1573 10.1124/mol.104.008730 15728261

[B17] Ellison T. I. Dowd D. R. MacDonald P. N. (2005). Calmodulin-dependent kinase IV stimulates vitamin D receptor-mediated transcription. Mol. Endocrinol. 19, 2309–2319 10.1210/me.2004-0382 15919723

[B18] Fernandes I. Bastien Y. Wai T. Nygard K. Lin R. Cormier O. (2003). Ligand-dependent nuclear receptor corepressor LCoR functions by histone deacetylase-dependent and -independent mechanisms. Mol. Cell. 11, 139–150 10.1016/S1097-2765(03)00014-5 12535528

[B19] Fischer J. Wang T.-T. Kaldre D. Rochel N. Moras D. White J. H. (2012). Synthetically accessible non-secosteroidal hybrid molecules combining vitamin D receptor agonism and histone deacetylase inhibition. Chem. Biol. 19, 963–971 10.1016/j.chembiol.2012.05.024 22921063

[B20] Fondell J. D. Ge H. Roeder R. G. (1996). Ligand induction of a transcriptionally active thyroid hormone receptor coactivator complex. Proc. Natl. Acad. Sci. U.S.A. 93, 8329–8333 10.1073/pnas.93.16.8329 8710870 PMC38670

[B21] Fraga R. Zacconi F. Sussman F. Ordóñez-Morán P. Muñoz A. Huet T. (2012). Design, synthesis, evaluation, and structure of vitamin D analogues with furan side chains. Chemistry 18, 603–612 10.1002/chem.201102695 22162241

[B22] Freedman L. P. Towers T. L. (1991). DNA binding properties of the vitamin D_3_ receptor zinc finger region. Mol. Endocrinol. 5, 1815–1826 10.1210/mend-5-12-1815 1665202

[B23] Fujii S. Masuno H. Taoda Y. Kano A. Wongmayura A. Nakabayashi M. (2011). Boron cluster-based development of potent nonsecosteroidal vitamin D receptor ligands: direct observation of hydrophobic interaction between protein surface and carborane. J. Am. Chem. Soc. 133, 20933–20941 10.1021/ja208797n 22066785

[B24] Fujiki R. Kim M.-S. Sasaki Y. Yoshimura K. Kitagawa H. Kato S. (2005). Ligand-induced transrepression by VDR through association of WSTF with acetylated histones. EMBO J. 24, 3881–3894 10.1038/sj.emboj.7600853 16252006 PMC1283952

[B25] Guzey M. Takayama S. Reed J. C. (2000). BAG1L enhances trans-activation function of the vitamin D receptor. J. Biol. Chem. 275, 40749–40756 10.1074/jbc.M004977200 10967105

[B26] Härd T. Kellenbach E. Boelens R. Maler B. A. Dahlman K. Freedman L. P. (1990). Solution structure of the glucocorticoid receptor DNA-binding domain. Science 249, 157–160 10.1126/science.2115209 2115209

[B27] Hong H. Kohli K. Garabedian M. J. Stallcup M. R. (1997). GRIP1, a transcriptional coactivator for the AF-2 transactivation domain of steroid, thyroid, retinoid, and vitamin D receptors. Mol. Cell. Biol. 17, 2735–2744 9111344 10.1128/mcb.17.5.2735 PMC232124

[B28] Hourai S. Fujishima T. Kittaka A. Suhara Y. Takayama H. Rochel N. (2006). Probing a water channel near the A-ring of receptor-bound 1 α,25-dihydroxyvitamin D_3_ with selected 2 α-substituted analogues. J. Med. Chem. 49, 5199–5205 10.1021/jm0604070 16913708

[B29] Hourai S. Rodrigues L. C. Antony P. Reina-San-Martin B. Ciesielski F. Magnier B. C. (2008). Structure-based design of a superagonist ligand for the vitamin D nuclear receptor. Chem. Biol. 15, 383–392 10.1016/j.chembiol.2008.03.016 18420145

[B30] Hsieh J.-C. Sisk J. M. Jurutka P. W. Haussler C. A. Slater S. A. Haussler M. R. (2003). Physical and functional interaction between the vitamin D receptor and hairless corepressor, two proteins required for hair cycling. J. Biol. Chem. 278, 38665–38674 10.1074/jbc.M304886200 12847098

[B31] Huet T. Maehr H. Lee H. J. Uskokovic M. R. Suh N. Moras D. (2011). Structure–function study of gemini derivatives with two different side chains at C-20, Gemini-0072 and Gemini-0097. Med. Chem. Commun. 2:424 10.1039/c1md00059d 22180837 PMC3236830

[B32] Inaba Y. Nakabayashi M. Itoh T. Yoshimoto N. Ikura T. Ito N. (2010). 22S-butyl-1α,24R-dihydroxyvitamin D_3_: recovery of vitamin D receptor agonistic activity. J. Steroid Biochem. Mol. Biol. 121, 146–150 10.1016/j.jsbmb.2010.02.033 20211257

[B33] Inaba Y. Yoshimoto N. Sakamaki Y. Nakabayashi M. Ikura T. Tamamura H. (2009). A new class of vitamin D analogues that induce structural rearrangement of the ligand-binding pocket of the receptor. J. Med. Chem. 52, 1438–1449 10.1021/jm8014348 19193059

[B34] Ito M. Yuan C. X. Malik S. Gu W. Fondell J. D. Yamamura S. (1999). Identity between TRAP and SMCC complexes indicates novel pathways for the function of nuclear receptors and diverse mammalian activators. Mol. Cell. 3, 361–370 10.1016/S1097-2765(00)80463-3 10198638

[B35] Jian Y. Yan J. Wang H. Chen C. Sun M. Jiang J. (2005). Cyclin D_3_ interacts with vitamin D receptor and regulates its transcription activity. Biochem. Biophys. Res. Commun. 335, 739–748 10.1016/j.bbrc.2005.07.141 16105657

[B36] Kakuda S. Ishizuka S. Eguchi H. Mizwicki M. T. Norman A. W. Takimoto-Kamimura M. (2010). Structural basis of the histidine-mediated vitamin D receptor agonistic and antagonistic mechanisms of (23S)-25-dehydro-1α-hydroxyvitamin D_3_-26,23-lactone. Acta Crystallogr. D Biol. Crystallogr. 66, 918–926 10.1107/S0907444910020810 20693691

[B37] Kakuda S. Okada K. Eguchi H. Takenouchi K. Hakamata W. Kurihara M. (2008). Structure of the ligand-binding domain of rat VDR in complex with the nonsecosteroidal vitamin D_3_ analogue YR301. Acta Crystallogr. Sect F Struct. Biol. Cryst. Commun. 4, 970–973 10.1107/S1744309108026754 PMC2581693 18997319

[B38] Kashiwagi H. Ono Y. Shimizu K. Haneishi T. Ito S. Iijima S. (2011). Novel nonsecosteroidal vitamin D_3_ carboxylic acid analogs for osteoporosis, and SAR analysis. Bioorg. Med. Chem. 19, 4721–4729 10.1016/j.bmc.2011.07.001 21795053

[B39] Khorasanizadeh S. Rastinejad F. (2001). Nuclear-receptor interactions on DNA-response elements. Trends Biochem. Sci. 26, 384–390 10.1016/S0968-0004(01)01800-X 11406412

[B40] Kim J. Y. Son Y. L. Lee Y. C. (2009). Involvement of SMRT corepressor in transcriptional repression by the vitamin D receptor. Mol. Endocrinol. 23, 251–264 10.1210/me.2008-0426 19098224 PMC5419315

[B41] Kim M.-S. Fujiki R. Kitagawa H. Kato S. (2007). 1α,25(OH)_2_D_3_-induced DNA methylation suppresses the human *CYP27B1* gene. Mol. Cell. Endocrinol. 265–266, 168–173 10.1016/j.mce.2006.12.014 17250953

[B42] Kim S. Shevde N. K. Pike J. W. (2005). 1,25-Dihydroxyvitamin D_3_ stimulates cyclic vitamin D receptor/retinoid X receptor DNA-binding, co-activator recruitment, and histone acetylation in intact osteoblasts. J. Bone Miner. Res. 20, 305–317 10.1359/JBMR.041112 15647825

[B43] Krasowski M. D. Yasuda K. Hagey L. R. Schuetz E. G. (2005). Evolutionary selection across the nuclear hormone receptor superfamily with a focus on the NR1I subfamily (vitamin D, pregnane X, and constitutive androstane receptors). Nucl. Recept. 3:2 10.1186/1478-1336-3-2 16197547 PMC1262763

[B44] Lee M. S. Kliewer S. A. Provencal J. Wright P. E. Evans R. M. (1993). Structure of the retinoid X receptor α DNA binding domain: a helix required for homodimeric DNA binding. Science 260, 1117–1121 10.1126/science.8388124 8388124

[B45] Li Y. Lambert M. Xu H. (2003). Activation of nuclear receptors: a perspective from structural genomics. Structure (Camb.) 11, 741–746 10.1016/S0969-2126(03)00133-3 12842037

[B46] Liu Y. Y. Nguyen C. Peleg S. (2000). Regulation of ligand-induced heterodimerization and coactivator interaction by the activation function-2 domain of the vitamin D receptor. Mol. Endocrinol. 14, 1776–1787 10.1210/mend.14.11.0560 11075811

[B47] Lou X. Toresson G. Benod C. Suh J. H. Philips K. J. Webb P. (2014). Structure of the retinoid X receptor α-liver X receptor β (RXRα-LXRβ) heterodimer on DNA. Nat. Struct. Mol. Biol. 21, 277–281 10.1038/nsmb.2778 24561505

[B48] Lou Y.-R. Molnár F. Peräkylä M. Qiao S. Kalueff A. V. St-Arnaud R. (2010). 25-Hydroxyvitamin D_3_ is an agonistic vitamin D receptor ligand. J. Steroid Biochem. Mol. Biol. 118, 162–170 10.1016/j.jsbmb.2009.11.011 19944755

[B49] Maehr H. Rochel N. Lee H. J. Suh N. Uskokovic M. R. (2013). Diastereotopic and deuterium effects in gemini. J. Med. Chem. 56, 3878–3888 10.1021/jm400032t 23566225

[B50] Mahajan M. A. Samuels H. H. (2000). A new family of nuclear receptor coregulators that integrate nuclear receptor signaling through CREB-binding protein. Mol. Cell. Biol. 20, 5048–5063 10.1128/MCB.20.14.5048-5063.2000 10866662 PMC85955

[B51] Makishima M. Lu T. Xie W. Whitfield G. Domoto H. Evans R. (2002). Vitamin D receptor as an intestinal bile acid sensor. Science 296, 1313–1316 10.1126/science.1070477 12016314

[B52] Malik S. Guermah M. Yuan C.-X. Wu W. Yamamura S. Roeder R. G. (2004). Structural and functional organization of TRAP220, the TRAP/mediator subunit that is targeted by nuclear receptors. Mol. Cell. Biol. 24, 8244–8254 10.1128/MCB.24.18.8244-8254.2004 15340084 PMC515042

[B53] Malloy P. J. Pike J. W. Feldman D. (1999). The vitamin D receptor and the syndrome of hereditary 1,25-dihydroxyvitamin D-resistant rickets. Endocr. Rev. 20, 156–188 10204116 10.1210/edrv.20.2.0359

[B54] Marshall P. A. Hernandez Z. Kaneko I. Widener T. Tabacaru C. Aguayo I. (2012). Discovery of novel vitamin D receptor interacting proteins that modulate 1,25-dihydroxyvitamin D_3_ signaling. J. Steroid Biochem. Mol. Biol. 132, 147–159 10.1016/j.jsbmb.2012.05.001 22626544 PMC3408799

[B55] Masuno H. Ikura T. Morizono D. Orita I. Yamada S. Shimizu M. (2013). Crystal structures of complexes of vitamin D receptor ligand-binding domain with lithocholic acid derivatives. J. Lipid Res. 54, 2206–2213 10.1194/jlr.M038307 23723390 PMC3708370

[B56] Masuyama H. Hiramatsu Y. (2004). Involvement of suppressor for Gal 1 in the ubiquitin/proteasome-mediated degradation of estrogen receptors. J. Biol. Chem. 279, 12020–12026 10.1074/jbc.M312762200 14702340

[B57] Mizwicki M. T. Keidel D. Bula C. M. Bishop J. E. Zanello L. P. Wurtz J.-M. (2004). Identification of an alternative ligand-binding pocket in the nuclear vitamin D receptor and its functional importance in 1α,25(OH)_2_-vitamin D_3_ signaling. Proc. Natl. Acad. Sci. U.S.A. 101, 12876–12881 10.1073/pnas.0403606101 15326291 PMC516488

[B58] Molnár F. Matilainen M. Carlberg C. (2005). Structural determinants of the agonist-independent association of human peroxisome proliferator-activated receptors with coactivators. J. Biol. Chem. 280, 26543–26556 10.1074/jbc.M502463200 15888456

[B59] Molnár F. Peräkylä M. Carlberg C. (2006). Vitamin D receptor agonists specifically modulate the volume of the ligand-binding pocket. J. Biol. Chem. 281, 10516–10526 10.1074/jbc.M513609200 16478719

[B60] Molnár F. Sigüeiro R. Sato Y. Araujo C. Schuster I. Antony P. (2011). 1α,25(OH)(2)-3-Epi-Vitamin D_3_, a natural physiological metabolite of Vitamin D_3_: its synthesis, biological activity and crystal structure with its receptor. PLoS ONE 6:e18124 10.1371/journal.pone.0018124 21483824 PMC3069065

[B61] Nagy L. Schwabe J. (2004). Mechanism of the nuclear receptor molecular switch. Trends Biochem. Sci. 29, 317–324 10.1016/j.tibs.2004.04.006 15276186

[B62] Nakabayashi M. Tsukahara Y. Iwasaki-Miyamoto Y. Mihori-Shimazaki M. Yamada S. Inaba S. (2013). Crystal structures of hereditary vitamin D-resistant rickets-associated vitamin D receptor mutants R270L and W282R bound to 1,25-dihydroxyvitamin D_3_ and synthetic ligands. J. Med. Chem. 56, 6745–6760 10.1021/jm400537h 23944708

[B63] Nakabayashi M. Yamada S. Yoshimoto N. Tanaka T. Igarashi M. Ikura T. (2008). Crystal structures of rat vitamin D receptor bound to adamantyl vitamin D analogs: structural basis for vitamin D receptor antagonism and partial agonism. J. Med. Chem. 51, 5320–5329 10.1021/jm8004477 18710208

[B64] Nevado J. Tenbaum S. P. Aranda A. (2004). hSrb7, an essential human Mediator component, acts as a coactivator for the thyroid hormone receptor. Mol. Cell. Endocrinol. 222, 41–51 10.1016/j.mce.2004.05.003 15249124

[B65] Nishikawa J. Matsumoto M. Sakoda K. Kitaura M. Imagawa M. Nishihara T. (1993). Vitamin D receptor zinc finger region binds to a direct repeat as a dimer and discriminates the spacing number between each half-site. J. Biol. Chem. 268, 19739–19743 8396146

[B66] Norman A. W. Bouillon R. Farach-Carson M. C. Bishop J. E. Zhou L. X. Nemere I. (1993). Demonstration that 1 β,25-dihydroxyvitamin D_3_ is an antagonist of the nongenomic but not genomic biological responses and biological profile of the three A-ring diastereomers of 1 α,25-dihydroxyvitamin D_3_. J. Biol. Chem. 268, 20022–20030 8397195

[B67] Orlov I. Rochel N. Moras D. Klaholz B. P. (2012). Structure of the full human RXR/VDR nuclear receptor heterodimer complex with its DR3 target DNA. EMBO J. 31, 291–300 10.1038/emboj.2011.445 22179700 PMC3261568

[B68] Polly P. Herdick M. Moehren U. Baniahmad A. Heinzel T. Carlberg C. (2000). VDR-Alien: a novel, DNA-selective vitamin D_3_ receptor-corepressor partnership. FASEB J. 14, 1455–1463 10.1096/fj.14.10.1455 10877839

[B69] Rachez C. Lemon B. D. Suldan Z. Bromleigh V. Gamble M. Näär A. M. (1999). Ligand-dependent transcription activation by nuclear receptors requires the DRIP complex. Nature 398, 824–828 10.1038/19783 10235266

[B70] Reddy G. S. Rao D. S. Siu-Caldera M. L. Astecker N. Weiskopf A. Vouros P. (2000). 1α,25-dihydroxy-16-ene-23-yne-vitamin D_3_ and 1α,25-dihydroxy-16-ene-23-yne-20-epi-vitamin D_3_: analogs of 1α,25-dihydroxyvitamin D_3_ that resist metabolism through the C-24 oxidation pathway are metabolized through the C-3 epimerization pathway. Arch Biochem. Biophys. 383, 197–205 10.1006/abbi.2000.2074 11185554

[B71] Ren Y. Behre E. Ren Z. Zhang J. Wang Q. Fondell J. D. (2000). Specific structural motifs determine TRAP220 interactions with nuclear hormone receptors. Mol. Cell. Biol. 20, 5433–5446 10.1128/MCB.20.15.5433-5446.2000 10891484 PMC85995

[B72] Renaud J. Rochel N. Ruff M. Vivat V. Chambon P. Gronemeyer H. (1995). Crystal structure of the RAR-γ ligand-binding domain bound to all-trans retinoic acid. Nature 378, 681–689 10.1038/378681a0 7501014

[B73] Rochel N. Ciesielski F. Godet J. Moman E. Roessle M. Peluso-Iltis C. (2011). Common architecture of nuclear receptor heterodimers on DNA direct repeat elements with different spacings. Nat. Struct. Mol. Biol. 18, 564–570 10.1038/nsmb.2054 21478865

[B74] Rochel N. Hourai S. Moras D. (2010). Crystal structure of hereditary vitamin D-resistant rickets-Associated mutant H305Q of vitamin D nuclear receptor bound to its natural ligand. J. Steroid Biochem. Mol. Biol. 121, 84–87 10.1016/j.jsbmb.2010.04.008 20403435

[B75] Rochel N. Hourai S. Pérez-García X. Rumbo A. Mouriño A. Moras D. (2007). Crystal structure of the vitamin D nuclear receptor ligand binding domain in complex with a locked side chain analog of calcitriol. Arch. Biochem. Biophys. 460, 172–176 10.1016/j.abb.2007.01.031 17346665

[B76] Rochel N. Moras D. (2012). Crystal structure of a vitamin D_3_ analog, ZK203278, showing dissociated profile. Anticancer Res. 32, 335–339 22213324

[B77] Rochel N. Tocchini-Valentini G. Egea P. F. Juntunen K. Garnier J. M. Vihko P. (2001). Functional and structural characterization of the insertion region in the ligand binding domain of the vitamin D nuclear receptor. Eur. J. Biochem. 268, 971–979 10.1046/j.1432-1327.2001.01953.x 11179963

[B78] Rochel N. Wurtz J. Mitschler A. Klaholz B. Moras D. (2000). The crystal structure of the nuclear receptor for vitamin D bound to its natural ligand. Mol. Cell. 5, 173–179 10.1016/S1097-2765(00)80413-X 10678179

[B79] Saito H. Takagi K. Horie K. Kakuda S. Takimoto-Kamimura M. Ochiai E. (2013). Synthesis of novel C-2 substituted vitamin D derivatives having ringed side chains and their biological evaluation on bone. J. Steroid Biochem. Mol. Biol. 136, 3–8 10.1016/j.jsbmb.2013.02.004 23416104

[B80] Sawada D. Tsukuda Y. Saito H. Kakuda S. Takimoto-Kamimura M. Ochiai E. (2011). Development of 14-epi-19-nortachysterol and its unprecedented binding configuration for the human vitamin D receptor. J. Am. Chem. Soc. 133, 7215–7221 10.1021/ja201481j 21500802

[B81] Shaffer P. L. Gewirth D. T. (2002). Structural basis of VDR-DNA interactions on direct repeat response elements. EMBO J. 21, 2242–2252 10.1093/emboj/21.9.2242 11980721 PMC125986

[B82] Shaffer P. L. Gewirth D. T. (2004). Structural analysis of RXR-VDR interactions on DR3 DNA. J. Steroid Biochem. Mol. Biol. 89–90, 215–219 10.1016/j.jsbmb.2004.03.084 15225774

[B83] Shimizu M. Miyamoto Y. Takaku H. Matsuo M. Nakabayashi M. Masuno H. (2008). 2-Substituted-16-ene-22-thia-1α,25-dihydroxy-26,27-dimethyl-19-norvitamin D_3_ analogs: Synthesis, biological evaluation, and crystal structure. Bioorg. Med. Chem. 16, 6949–6964 10.1016/j.bmc.2008.05.043 18539034

[B84] Shindo K. Kumagai G. Takano M. Sawada D. Saito N. Saito H. (2011). New C15-substituted active vitamin D_3_. Org. Lett. 13, 2852–2855 10.1021/ol200828s 21539305

[B85] Stambolsky P. Tabach Y. Fontemaggi G. Weisz L. Maor-Aloni R. Siegfried Z. (2010). Modulation of the vitamin D_3_ response by cancer-associated mutant p53. Cancer Cell 17, 273–285 10.1016/j.ccr.2009.11.025 20227041 PMC2882298

[B86] Tagami T. Lutz W. H. Kumar R. Jameson J. L. (1998). The interaction of the vitamin D receptor with nuclear receptor corepressors and coactivators. Biochem. Biophys. Res. Commun. 253, 358–363 10.1006/bbrc.1998.9799 9878542

[B87] Thénot S. Henriquet C. Rochefort H. Cavailles V. (1997). Differential interaction of nuclear receptors with the putative human transcriptional coactivator hTIF1. J. Biol. Chem. 272, 12062–12068 10.1074/jbc.272.18.12062 9115274

[B88] Ting H.-J. Bao B.-Y. Hsu C.-L. Lee Y.-F. (2005). Androgen-receptor coregulators mediate the suppressive effect of androgen signals on vitamin D receptor activity. Endocrine 26, 1–9 10.1385/ENDO:26:1:001 15805579

[B89] Tocchini-Valentini G. Rochel N. Wurtz J. M. Mitschler A. Moras D. (2001). Crystal structures of the vitamin D receptor complexed to superagonist 20-epi ligands. Proc. Natl. Acad. Sci. U.S.A. 98, 5491–5496 10.1073/pnas.091018698 11344298 PMC33240

[B90] Tocchini-Valentini G. Rochel N. Wurtz J. Moras D. (2004). Crystal structures of the vitamin D nuclear receptor liganded with the vitamin D side chain analogues calcipotriol and seocalcitol, receptor agonists of clinical importance. Insights into a structural basis for the switching of calcipotriol to a receptor antagonist by further side chain modification. J. Med. Chem. 47, 1956–1961 10.1021/jm0310582 15055995

[B91] Toell A. Polly P. Carlberg C. (2000). All natural DR3-type vitamin D response elements show a similar functionality *in vitro*. Biochem. J. 352(Pt 2), 301–309 10.1042/0264-6021:3520301 11085922 PMC1221460

[B92] Towers T. L. Staeva T. P. Freedman L. P. (1999). A two-hit mechanism for vitamin D_3_-mediated transcriptional repression of the granulocyte-macrophage colony-stimulating factor gene: vitamin D receptor competes for DNA binding with NFAT1 and stabilizes c-Jun. Mol. Cell. Biol. 19, 4191–4199 10330159 10.1128/mcb.19.6.4191 PMC104378

[B93] Umesono K. Murakami K. K. Thompson C. C. Evans R. M. (1991). Direct repeats as selective response elements for the thyroid hormone, retinoic acid, and vitamin D_3_ receptors. Cell 65, 1255–1266 10.1016/0092-8674(91)90020-Y 1648450 PMC6159884

[B94] van Driel M. Koedam M. Buurman C. J. Roelse M. Weyts F. Chiba H. (2006). Evidence that both 1α,25-dihydroxyvitamin D_3_ and 24-hydroxylated D_3_ enhance human osteoblast differentiation and mineralization. J. Cell. Biochem. 99, 922–935 10.1002/jcb.20875 16741965

[B95] Vanhooke J. L. Benning M. M. Bauer C. B. Pike J. W. DeLuca H. F. (2004). Molecular structure of the rat vitamin D receptor ligand binding domain complexed with 2-carbon-substituted vitamin D_3_ hormone analogues and a LXXLL-containing coactivator peptide. Biochemistry 43, 4101–4110 10.1021/bi036056y 15065852

[B96] Vanhooke J. L. Tadi B. P. Benning M. M. Plum L. A. DeLuca H. F. (2007). New analogs of 2-methylene-19-nor-(20S)-1,25-dihydroxyvitamin D_3_ with conformationally restricted side chains: evaluation of biological activity and structural determination of VDR-bound conformations. Arch. Biochem. Biophys. 460, 161–165 10.1016/j.abb.2006.11.029 17227670

[B97] Verlinden L. Verstuyf A. Eelen G. Bouillon R. Ordóñez-Morán P. Larriba M. J. (2011). Synthesis, structure, and biological activity of des-side chain analogues of 1α,25-dihydroxyvitamin D_3_ with substituents at C18. ChemMedChem 6, 788–793 10.1002/cmdc.201100021 21520419

[B98] Watkins R. Wisely G. Moore L. Collins J. Lambert M. Williams S. (2001). The human nuclear xenobiotic receptor PXR: structural determinants of directed promiscuity. Science 292, 2329–2333 10.1126/science.1060762 11408620

[B99] Whitfield G. K. Dang H. T. L. Schluter S. F. Bernstein R. M. Bunag T. Manzon L. A. (2003). Cloning of a functional vitamin D receptor from the lamprey (Petromyzon marinus), an ancient vertebrate lacking a calcified skeleton and teeth. Endocrinology 144, 2704–2716 10.1210/en.2002-221101 12746335

[B100] Winn M. D. Ballard C. C. Cowtan K. D. Dodson E. J. Emsley P. Evans P. R. (2011). Overview of the CCP4 suite and current developments. Acta Crystallogr. D Biol. Crystallogr. 67, 235–242 10.1107/S0907444910045749 21460441 PMC3069738

[B101] Yanagisawa J. Yanagi Y. Masuhiro Y. Suzawa M. Watanabe M. Kashiwagi K. (1999). Convergence of transforming growth factor-β and vitamin D signaling pathways on SMAD transcriptional coactivators. Science 283, 1317–1321 10.1126/science.283.5406.1317 10037600

[B102] Yoshimoto N. Sakamaki Y. Haeta M. Kato A. Inaba Y. Itoh T. (2012). Butyl pocket formation in the vitamin D receptor strongly affects the agonistic or antagonistic behavior of ligands. J. Med. Chem. 55, 4373–4381 10.1021/jm300230a 22512505

[B103] Zhang J. Chalmers M. J. Stayrook K. R. Burris L. L. Wang Y. Busby S. A. (2011). DNA binding alters coactivator interaction surfaces of the intact VDR-RXR complex. Nat. Struct. Mol. Biol. 18, 556–563 10.1038/nsmb.2046 21478866 PMC3087838

